# Assessing the utility of statistical downscaling for subseasonal temperature forecasts

**DOI:** 10.1038/s41598-026-45067-2

**Published:** 2026-03-26

**Authors:** Eren Duzenli, Jaume Ramon, Verónica Torralba, Sam Pickard, Ángel G. Muñoz, Dragana Bojovic

**Affiliations:** Earth Sciences Department, Barcelona Supercomputing Center (BSC), Barcelona, Spain

**Keywords:** Statistical downscaling, Subseasonal prediction, Temperature extremes, Skill assessment, Method comparison, Weather regimes, Climate sciences, Environmental sciences

## Abstract

Subseasonal temperature forecasts can guide timely action against heat risks, but their coarse resolution limits regional usefulness. We apply a subseasonal downscaling framework to benchmark 27 statistical methods, including configurations of bias correction, linear and logistic regression, and analogs, to assess how they transfer forecast skill from coarse to local resolution at the weekly scale. As a test case, retrospective forecasts from CFSv2 (Climate Forecast System version 2; ~100 km) are downscaled to ~ 5 km for initializations issued one to four weeks before each target week of the Paris 2024 Olympics. Skill is assessed with the Brier Skill Score at the 10th and 90th percentiles for extremes. We also test whether incorporating atmospheric patterns adds value to downscaling. Methods are implemented with both daily and weekly data to examine the role of temporal resolution. Results show that, although most methods successfully transfer CFSv2 skill to higher resolution, method choice remains critical, as some degrade skill while others enhance it. Methods incorporating atmospheric patterns show promise at longer lead times when the relevant pattern, climatologically a key driver of heat in the target week, is well predicted. At the subseasonal scale, downscaling with weekly predictors outperforms that based on daily predictors.

**Supplementary Information:**

The online version contains supplementary material available at 10.1038/s41598-026-45067-2.

## Introduction

Apart from raising average global temperature values, climate change also tends to increase the frequency and intensity of extreme events, such as heatwaves, jeopardizing public health^1–3^. These heatwaves can be particularly hazardous during summer sporting events like the Summer Olympic and Paralympic Games where risks pertain not just to athletes who risk exertional heat stress while training and competing outdoors during the day, but also to event staff and spectators^4^. To manage the risks of heat stress for the high numbers of participants, event organizers need localised information about temperature behavior with adequate temporal resolution and lead time to make timely decisions and implement compensatory measures. Current information systems include short-range weather forecasts up to approximately 10 days ahead^5^, seasonal forecasts that present data on a monthly or seasonal scale^6–10^. The gap between weather and seasonal forecasts is covered by the subseasonal forecasts, which can provide weekly predictions that extend from two weeks to two months into the future^11–14^. Timely and adequately scaled subseasonal forecasts can help support decision making for events such as the Paris 2024 Olympic and Paralympic Games.

Although subseasonal predictions remain more sensitive to atmospheric initial conditions than seasonal predictions, predictive skill in subseasonal forecasts primarily comes from components of the climate system that evolve more slowly than those important for short-range weather forecasts^15–17^. Notable examples are the land surface and ocean, the states of which can be crucial for subseasonal prediction quality. Relatedly, the Madden–Julian Oscillation (MJO) is often considered the main source of global subseasonal predictive skill, especially in the tropics and extratropics^18^ but other climate processes such as soil moisture, oceanic state, sea ice interaction, upper-level atmospheric interactions, and tropical and extratropical interactions also play significant roles in the skill of forecasts at subseasonal time scales^19–22^. Temperature predictions from subseasonal forecast products thus have the potential to provide weekly information about heat-related issues 4 to 6 weeks in advance.

Regarding spatial scale, global climate models (GCMs) that provide subseasonal predictions operate at a relatively coarse resolution. The highest-resolution global subseasonal-to-seasonal climate prediction model has a spatial resolution of 0.25°, while most models have even coarser resolutions^23^ owing to the substantial computational resources that would be required for higher-resolution grids. However, regional climates are influenced not only by global circulation but also by local-scale processes, and the coarse-scale grids in GCMs cannot resolve such intricate local-scale interactions^24–27^. Here, statistical downscaling serves as a valuable and computationally economic tool, supplying high-resolution regional climate information to users by blending the coarse resolution outputs of GCMs with local-scale hydroclimatological observations. Either the target variable from the GCM (as in model output statistics approaches; MOS) or other large-scale variables, such as mean sea level pressure (SLP), geopotential height, or weather regimes (WRs) (as in the perfect prognosis approach; PP), can be used as a predictor in statistical downscaling^28–32^.

Plausible statistical downscaling methodologies at subseasonal timescales are similar to those at other timescales, including various regression and deep learning algorithms as well as simple bias adjustment merged with interpolation techniques. In recent years, several studies have focused on downscaling subseasonal climate predictions through statistical methods^33–35^. However, the literature remains limited, and many research questions are still unexplored. Importantly, selecting the most appropriate statistical approach to translate coarse GCM information to high resolution, while preserving and ideally enhancing quality at reasonable computational cost, is crucial. Contrary to the common assumption, recent studies have shown that downscaling tends to considerably contribute to the final uncertainty of the decision-making outcomes, and that in general the model predictive skill does not necessarily increase^36,37^. Better understanding the conditions under which downscaling improves the utility of information at subseasonal scale, compared to the coarse-resolution GCM output, in decision making frameworks requires additional studies, like the one presented in this paper.

We aim to evaluate the potential of statistical downscaling for transferring subseasonal temperature forecasts from coarse (~ 100 km) to high (~ 5 km) resolution through a comparative assessment of methods. The Paris 2024 Olympics is used as the case study due to its practicality and clear relevance. However, this work is not intended to deliver an operational forecast product for the event; instead, it is designed to explore insights into how statistical downscaling can impact forecast quality at subseasonal timescales, including the influence of large-scale predictors, the downscaling workflow and techniques, and to indicate the potential usefulness of the resulting information for extreme temperature planning. In this context, the emphasis is not on developing a system that consistently outperforms climatology, but on examining whether there are cases where downscaling increases forecast skill relative to the raw GCM forecasts and, when improvements occur, understanding the mechanisms that may explain them. Using MOS and PP, we identify and test a pool of 27 downscaling configurations drawn from four method families: bias correction, linear regression, logistic regression, and analogs. For each configuration, we produce downscaled forecasts one, two, three, and four weeks before the target week. The extended-window approach, detailed in the section “Downscaling framework and data processing”, is utilized to better represent the weekly temperature climatology and improve the quality of the statistical downscaling products. We also address specific questions relevant to the development of high-quality subseasonal climate products:

- Can GCM skill be improved through statistical downscaling? If so, what is the source of this enhancement?
- Do the methods using the PP approach provide better performance compared to methods using the MOS approach?
- Do models built with daily data perform better than those built with weekly data?
- Do the best methods depend on the lead time, the evaluation metric or the target week?

## Materials and methods

### Data

We used daily and weekly mean temperature together with daily mean SLP. Anomalies were computed relative to each dataset’s own 1999–2018 climatology over the hindcast period, and all subsequent steps were based on these anomalies. Only temperature anomalies were downscaled, while SLP anomalies were used as large-scale predictors for temperature downscaling through PP approach and for obtaining WRs (Section “Analogs-hybrid”). When analyzing influences of the large-scale circulations on local conditions, geopotential height and SLP are the most commonly used variables^38–44^. In this study, we selected SLP over geopotential height as the large-scale predictor and WR generator, following Hartmann et al. (2013)^45^. Their results indicate that SLP is less influenced by climate change than geopotential height and may therefore provide a more temporally consistent basis for WR identification over the study period.

Subseasonal predictions were obtained from an ensemble-based system, the Climate Forecast System Version 2 (CFSv2^46^), developed by the National Centers for Environmental Prediction (NCEP). We chose the CFSv2 subseasonal product primarily due to the availability of a consistent hindcast archive and its relevance for subseasonal prediction research. Its real-time forecasting capability represents an additional advantage in view of potential future operational applications. At a spatial resolution of approximately 100 km, 45-day retrospective forecasts (i.e. hindcasts) are available starting from January 1999. Time resolution of the CFSv2 output is 6-hourly with hindcast members formed by initiating the model four times per day. This lagged approach produces four ensemble members per day, obtained from model initiations at each cycle (i.e., 0000, 0600, 1200, and 1800 UTC).

The single-level Copernicus European Regional ReAnalysis dataset (CERRA^47^) was used as the reference dataset. With approximately 5 km spatial resolution for Europe, it is available from 1984 to the present at a 3-hourly temporal scale. Still, considering the hindcast availability, the study period consisted of 20 years, spanning from 1999 to 2018. CERRA is based on observational datasets, the ERA5 reanalysis dataset for initial and lateral boundary conditions and prior estimates of the atmospheric and physiographic conditions. We chose CERRA rather than ERA5 because of the former’s higher spatial resolution (5 km). However, since CERRA does not provide data for a portion of the North Atlantic, we used SLP data from ERA5 when determining WRs (Section “Analogs-hybrid”).

Before using CERRA as the reference for the downscaling methods, we evaluated its consistency with the E-OBS dataset^48^, a widely used gridded observational product. The results indicated a high level of agreement between the two datasets. Based on grid-point daily mean temperature comparisons between CERRA and E-OBS, correlation values across Target Weeks 1–3 show 10th–90th percentile ranges of 0.93–0.97, while the corresponding RMSE ranges are 0.74–1.05 °C, 0.69–1.04 °C, and 0.73–1.03 °C, respectively. Additional details of this evaluation, along with further analyses of the CERRA data over the study area and period, are provided in the supplementary material to support the study with background information.

### Downscaling framework and data processing

**Fig. 1:**
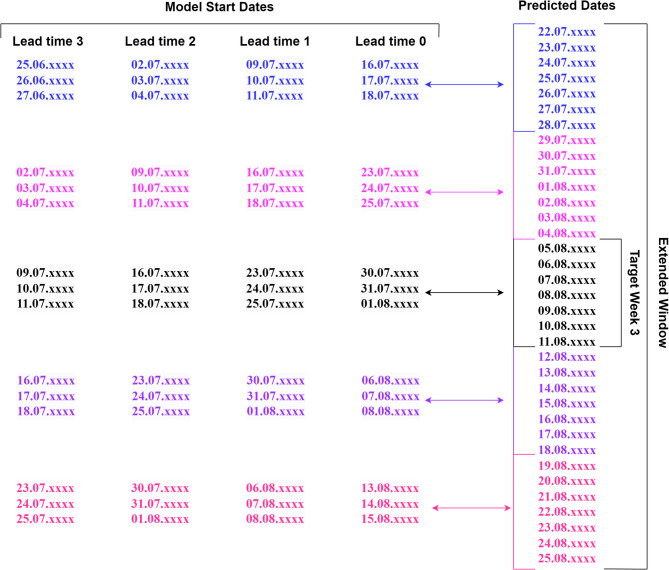
CFS-v2 start dates according to lead times and the time series obtained with the extended window. This is an example demonstration for Target Week 3, but the same process has been applied to the other target weeks as well. For daily analysis of Target Week 3, 35 days per year between 22.07.xxxx and 25.08.xxxx were used for downscaling and skill calculation processes (a total of 700 data points). In weekly analyses, weekly averages are obtained from the daily data and the processes are carried out with 5 data points per year (a total of 100).

Having gathered the data, downscaling was performed separately on the retrospective forecasts for each of the three target weeks of the Paris 2024 Olympics (Target Week 1: July 22–28, Target Week 2: July 29–August 4, Target Week 3: August 5–11). Furthermore, the downscaling was carried out for the target weeks at four different lead times (0, 1, 2, 3 weeks). Lead times 0, 1, 2, and 3 refer to the downscaling of model data whose nominal start date is 1, 2, 3, and 4 weeks prior to the mid-day of the corresponding target week (Fig. 1).

Both reference and hindcast data were averaged from their native time resolutions into daily or weekly values with respect to the downscaling methodology being analysed. In the scope of our downscaling framework:

1. We used the extended-window concept to include not only the days within the target week but also the days within the two weeks before and after in the downscaling and skill assessment (Fig. 1). In other words, during the downscaling process, parameter and correction function estimations, as well as analog searches, were performed using the dataset brought together through the extended window. Similarly, skill score computations and the resulting statistical significance estimations were also based on the same extended-window dataset. This means that we used 35 data points per year instead of 7, resulting in a total of 700 data points over the 20-year period for daily-averaged methods, and 100 data points for weekly-averaged methods. Besides increasing the amount of data, the extended-window approach allowed us to consider the influence of neighboring weeks on the climatology of the target week, which is crucial in subseasonal climate prediction^17^.
2. As stated in the section Data, the CFSv2 model produces four ensemble members per day. Accordingly, we included forecasts from the models launched within 2 days prior to each model initialization day to increase the number of ensemble members for the relevant target weeks (Fig. 1). These members are considered under the assumption of exchangeability, treating them without distinction by initialization time, as this ensures a coherent and practical treatment across methods where ensemble spread and central tendencies are of primary interest.
3. For each nominal start date of the model, the data for the first three days were omitted. This accounts for the fact that the skill for the first days comes from the influence of the atmospheric initial conditions, leading to an overestimation of skill for the first lead time week due to the better performance of weather forecasts compared to subseasonal predictions^49^. For instance, for Target Week 3, whose first day is the 5th of August, removing the first three days leads to selecting the model initialization on August 1 st (lead time 0). Also considering the second point above, for lead time 0, the 12 ensemble-member predictions used for the target week of 5 to 11 August are obtained from simulations launched on 30 July, 31 July, and 1 August (four members per day). The CFS-v2 initialisation dates of Target Week 3 for each of the lead times are summarised in Fig. 1.

All steps above were carried out to compensate for the potential limitations of having fewer ensemble members per day and relying on a relatively short study period (i.e., 20 years) in the hindcast data. This limitation is further discussed in Leutbecher et al. (2019)^50^ and Manrique-Suñén et al. (2020)^17^.

To summarize the training setup, downscaling models were trained separately for each target week and each lead time described above. Predictors derived from CFSv2 hindcasts at a given lead time were paired with CERRA reference fields at the same valid dates within the extended window, ensuring temporal consistency between the retrospective forecast and observation. Within the leave-one-year-out cross-validation framework, models were trained over the 1999–2018 period excluding the target year, using data from the corresponding extended window. All data from the excluded year were then used for independent prediction. This process was repeated for each year in the study period, resulting in lead-time-specific relationships estimated independently for each target week.

### Downscaling methods

The two main statistical downscaling approaches, MOS and PP, are commonly distinguished by the source and use of predictors^29^. In MOS, GCM outputs are used as predictors, and a statistical relationship is built directly between these modelled predictors and the local-scale predictand (e.g., observed temperature). While the predictor does not have to be the same variable as the predictand, MOS is often applied in such a way (e.g., model temperature to observed temperature), particularly for bias correction purposes. As a result, its skill becomes highly dependent on the GCM’s capacity to reproduce the local-scale variable in question. In contrast, PP uses observational or reanalysis data to derive the relationship between large-scale predictors (e.g., geopotential height, sea-level pressure) and local observations. This relationship is then applied to GCM predictors to generate the downscaled variable. Thus, PP separates model simulation quality from statistical training, potentially increasing robustness when GCMs poorly simulate local-scale processes.

This study tested 27 different methodologies under four groups of statistical downscaling methods: analogs, bias correction, linear regression and logistic regression (Table 1). For all groups except analogs, the coarse-resolution dataset (i.e. CFSv2) was first disaggregated using either bicubic or conservative interpolation to match the spatial resolution of the reference dataset (i.e. CERRA). The main reason for selecting bicubic and conservative interpolation methods is based on a pre-sensitivity study. Before applying any correction, bicubic, bilinear, conservative, second-order conservative, inverse distance weighting, and nearest neighbor interpolation methods were utilized to interpolate coarse-scale data to a higher resolution for some specific cases. The skills of the interpolated data were then compared. Overall, the bicubic and conservative methods provided slightly better results than the other methods.

**Table 1:** A total of 27 methods corresponding to the combinations of the characteristics listed in the columns below were tested. For example, for the analog methods, all possible combinations of number of analogs, temporal scale, and downscaling approach were tested. This continues in the same way for the other columns as well. Downscaling operations were executed using the CSDownscale^51^ and SUNSET^52^ R packages.

	Analogs	Bias Correction	Linear regression	Logistic regression
Interpolation	-	Bicubic, Conservative	Bicubic, Conservative	Bicubic
Downscaling technique	-	Evmos	Simple, 9nn^‡^	Ensemble mean, Ensemble mean and standard deviation
Downscaling approach	MOS, PP, Hybrid^†^	MOS	MOS	MOS
Temporal scale	Daily, Weekly*	Weekly	Daily, Weekly	Daily, Weekly
Analog number	1, 5, 15	-	-	-

^†^Two Hybrid configurations were used, differing by whether EOF prefiltering was applied.

*Weekly data was used only with the MOS approach, while daily data is used with the MOS, PP, and hybrid approaches.

^‡^Since no pre-interpolation is required, two 9nn methods were built, one with weekly and one with daily data.

After interpolation, we built the statistical relationship between the model and observation data. For bias correction, linear regression, logistic regression methods, downscaling was conducted locally, meaning the relationship is built and applied independently in each grid point. The only exception was the ‘nine nearest neighbors multiple linear regression method’ (9nn), which is a non-local method that does not need a prior interpolation of data because it uses the neighboring grid points in the coarse grid to build the statistical relationship. The MOS approach was used for bias correction, and linear and logistic regression methods. In the analogs methods, we tested both PP and hybrid approaches in addition to the MOS approach. We will address the potential added value of using large-scale variables in the ''Results and discussion'' section.

Except for the logistic regression approach, which uses predictors based on the ensemble mean or standard deviation, the downscaling methods explained in the following sections were applied separately to each ensemble member.

#### Bias correction

We used the variance inflation (evmos) technique^53^ as the bias correction method. This corrects both the mean bias and the variance bias of the input data based on the reference dataset. Two interpolation schemes (bicubic and conservative) were tested to assess their influence on the final downscaled skill. Weekly data were utilised for the variance inflation techniques.

#### Linear regression

We tested three different linear regression techniques. The first two techniques involve simple linear regression, whereas the third technique was the 9nn method with principal component (PC) pre-filtering. For the 9nn method, we first identified the nine nearest neighboring grid points in the coarse-resolution GCM data for each observation grid point. Then, PC analysis was applied to the predictors (i.e. the nine neighboring grids) before building the model for the corresponding reference grid, to address possible collinearity issues. PCs explaining 95% of the variability were used as predictors to establish multiple linear regression models at the reference grid points. As done for bias correction, we tested bicubic and conservative interpolation methods for the simple linear regression (no interpolation is used in 9nn) and for both the simple and the 9nn techniques we tested the impact on model performance of using daily and weekly data.

#### Logistic regression

The logistic regression method uses a sigmoid function to relate ensemble mean of the anomalies of the coarse scale forecasts directly to probabilities of observing specific categories^54^. In this study, we defined categories for extremes, i.e. rare events which are located in the upper and lower tails (above the 90th percentile and below the 10th percentile, respectively) of the climatological distribution. We tested both bicubic and conservative interpolations to disaggregate the coarse resolution data; however, bicubic interpolation consistently yielded better results with logistic regression methods compared to the conservative interpolation. Therefore, only the results obtained from bicubic interpolation are presented in this paper. We used two different logistic regression techniques, which are distinguished by their predictors. In the ensemble mean technique, the ensemble mean of the CFSv2 hindcast for each time step was calculated and used as the predictor to estimate the categorical values of the observations. For the ensemble mean and standard deviation, we employed a multi logistic regression that uses the standard deviations of the ensemble member time steps as an additional predictor, along with the ensemble mean. All the logistic regression techniques were tested with daily and weekly data.

#### Analogs

The analogs method^55^ is one of the simplest statistical downscaling methods, which basically involves searching for analog(s) of the model fields (e.g. CFSv2) in the historical records of a reference dataset (e.g. CERRA). Many studies have explored various analog approaches for both the calibration and the downscaling of climate prediction data^36,56–58^. The analog methods used in this study are described in detail in the following subsections. In this work, the selection of analogs is based on Euclidean distance. We evaluated three approaches (MOS, PP, and hybrid) and for each, we examined varying numbers of analogs (i.e. 1, 5, 15). For cases selecting more than 1 analog, the final downscaled products were calculated as the weighted mean of the 5 or 15 most analogous fields. The weights were calculated inversely proportional to the Euclidean distance values of these analog fields. We used daily and weekly data to construct analogs models, except for the methods including large-scale variables, where only daily data were used.

##### Analogs-MOS

In this approach, the most similar atmospheric field(s) for the variable to be downscaled is selected from the historical observations of the same variable (in this case, temperature). For each specific time step of the CFSv2 temperature fields, we calculated the Euclidean distance between the field and each time step of the CERRA temperature fields. The CERRA dataset time step that resulted in the smallest Euclidean distance was then assigned as the downscaled temperature data for the corresponding CFSv2 time step. This process was repeated for each time step of the CFSv2 data.

##### Analogs-PP

The Analogs-PP approach examines large-scale variables to identify the most analogous time step. Once the most similar time step was determined from the large-scale fields, the local-scale field in the observation for the corresponding time step was then retrieved and marked as the downscaled data of the time step in question.

We used the SLP field over the North Atlantic region as the large-scale variable, aiming to leverage the potential influence of the North Atlantic SLP on the summer temperature of the Paris region. However, instead of using SLP data from the traditional North Atlantic region covering 27°–81°N and 85.5°W–45°E^59–61^, we used data from a smaller region (30°W – 25°E, 30°N – 65°N) as Garrido-Perez et al. (2020)^62^ showed that this smaller area better represents the influence of SLP circulations in the North Atlantic on some surface variables in western Europe. Using the smaller region also significantly reduced the computational cost.

##### Analogs-hybrid

The Analogs-hybrid approach uses the WRs to downscale the temperature data. WRs were derived from the ERA5-SLP anomalies over the smaller North Atlantic region introduced in the previous section. The spatial patterns of the WR field with the minimum Euclidean distance for each time step of the CFSv2 SLP field was then identified and assigned to the corresponding CFSv2 time step. We then searched for the analogs among the temperature variables belonging to the same WR. For instance, if the CFSv2 temperature data for a certain time step belonged to WR1, we only searched for analogs for this field among the temperature values of the reanalysis data within WR1. The main purposes here were to apply a pre-filtering that addresses the driving influence of the SLP anomalies on the temperature data, and to select the temperature analogs from the reanalysis data that show similarity to the relevant GCM time step in terms of the atmospheric circulation patterns represented by WRs.

The *k*-means algorithm^63^ was used to acquire the driving WRs. This algorithm clusters the points (i.e. SLP fields in our case) into *k* groups. The objective function of the algorithm is to minimize the sum of squares between the individual points and their assigned cluster centers. Due to the low variability of the atmospheric circulation over the Euro-Atlantic in summer, there is no consensus on the optimal number of clusters (i.e. the value of *k*) in summer WR studies^64^. Therefore, we carried out preliminary tests of different values to determine the number of clusters to be used in the study (i.e. *k* = 3, 4, 5, 6). We found that when 5 or 6 clusters were selected, some of the WRs are significantly correlated, indicating similar spatial patterns and making it difficult to distinguish their effects from one another. Therefore, we omitted *k* = 5 and *k* = 6 cases from the study. We then explored the difference between *k* = 3 and *k* = 4, via downscaling for several specific cases and found no significant difference between the downscaling outputs. Hence, following *Cassou* et al. (2005)^65^, who previously investigated the impact of WRs on the heatwaves over France and suggested that the ideal number of the summer WRs is 4 for the region, we selected *k* = 4.

We also set out to investigate the impact of the common practice of applying a pre-filtering process before the *k*-means clustering step to reduce the spatiotemporal dimensions of the data used to obtain WRs^39,58,66–69^. Correspondingly, the WRs were calculated both with and without the empirical orthogonal function (EOF) pre-filtering. The pre-filtering process aims to extract leading EOF modes that explain a certain amount of variability in the dataset, increase spatial interpretability, and reduce redundant complexity and noise in the data, thereby highlighting the main variation modes. Here, we applied the *k*-means algorithm after obtaining the leading EOFs explaining 95% of the variability in the SLP data. The WRs were calculated individually for each target week using the daily data within the extended window described in the section Data (i.e. 700 daily data per target week were used to determine the WRs).

Composite maps illustrating four WRs, produced with or without EOF pre-filtering for each target week, are presented in Fig. S3. No further analysis of WRs was included in this study since the only purpose of including WRs was to classify the CFSv2 and CERRA temperature variables. Nonetheless, a visual comparison between the WRs with and without EOF-prefiltering (Fig. S3) shows that in general there are no major differences, which contrasts with the usual recommendation of always conducting an EOF-prefiltering before computing the WRs. We attribute it to this particular case under study not having a lot of atmospheric noise, which is consistent with the enhanced stability exhibited by the regional atmospheric circulation patterns during the summer in this region. Still, the impact of the EOF filtering arises for Target Week 2. For example, the WR4 based on filtered EOFs shows negative SLP anomalies over Iceland and positive SLP anomalies over western Europe. This spatial distribution of the SLP anomalies is not found when the EOF filtering is not applied. Hence, this illustrates the importance of the EOF filtering step for the calculation of the WRs.

### Skill assessment

The probabilistic skill of the downscaled subseasonal forecasts was measured using the Brier Skill Score − 10% (BSS10), and Brier Skill Score − 90% (BSS90^70^) BSS10 and BSS90 aim to discern whether forecasts can anticipate rare (extreme) events that fall below the 10th percentile and above the 90th percentile of the climatological distribution, respectively. Brier Scores account for both reliability and resolution, where low reliability results in large errors, and poor resolution means the forecasts fail to confidently distinguish events. The metrics were calculated for each grid point separately using CERRA as the reference data set. Regardless of whether the downscaling method was carried out for daily or weekly data, the skill assessment was carried out only at the weekly scale to align with subseasonal model outputs. To this end, the outputs of the daily data-driven methods were averaged to the weekly scale prior to the skill calculation. These skill scores range between −∞ and + 1 and use climatological prediction as a reference. Climatological prediction assumes that the probability of being in the lowest/highest 10% is 10%, and the probability of not being in the lowest/highest 10% is 90%. Skill scores > 0 suggest the downscaled product is a better prediction than the climatological forecast (i.e. it is “skillful”). The ranking of the downscaling methods was based upon the skill estimates of the downscaled subseasonal predictions. Lastly, when calculating the skill metrics for CFSv2 (i.e. non-downscaled predictions), CERRA data was upscaled to match the resolution of the CFSv2 data.

In order to assess the generalizability of the skill metrics and eliminate random effects, the statistical significance of the skill scores was tested at the 95% confidence level. The test was conducted to determine whether the CFSv2 predictions or downscaled products were significantly better than the climatological prediction. The random walk test, a rank-based significance test suggested by^71^, was utilized. This approach evaluates the significance of BSS10 and BSS90 by applying the random walk test to the Brier Score − 10% (BS10), and Brier Score − 90% (BS90) time series, respectively. The test assesses whether the number of instances in a time series where the downscaled data outperforms the reference prediction is statistically significant at the 95% confidence level. Therefore, with this approach, even if the skill score is small, a product can still be significantly better than another because the evaluation focuses on the frequency of better performance rather than its magnitude^3^. To control for the multiple testing problem, we applied the false discovery rate (FDR) correction^72^, using αFDR = 0.1, which approximately corresponds to a global significance level of 0.05.

Furthermore, fair versions of the skill scores were calculated instead of the actual values. The fair version is used when comparing different models (or versions of the same model) with varying ensemble sizes^73,74^. It can be considered a potential skill score that would emerge with a sufficiently large ensemble size. In our study, as explained in the section “Downscaling framework and data processing”, the ensemble size of the predictions was extended by incorporating ensemble members from the previous two days and applying the extended window approach, aiming to address the limited number of ensemble members and obtain a better estimation of climatology and skill. Therefore, we calculated the fair skill scores of the downscaling methods, which aim to provide a skill score representative of products with a sufficiently large ensemble size.

## Results and discussion

### Skill comparison of the downscaled and coarse subseasonal forecasts

**Fig. 2:**
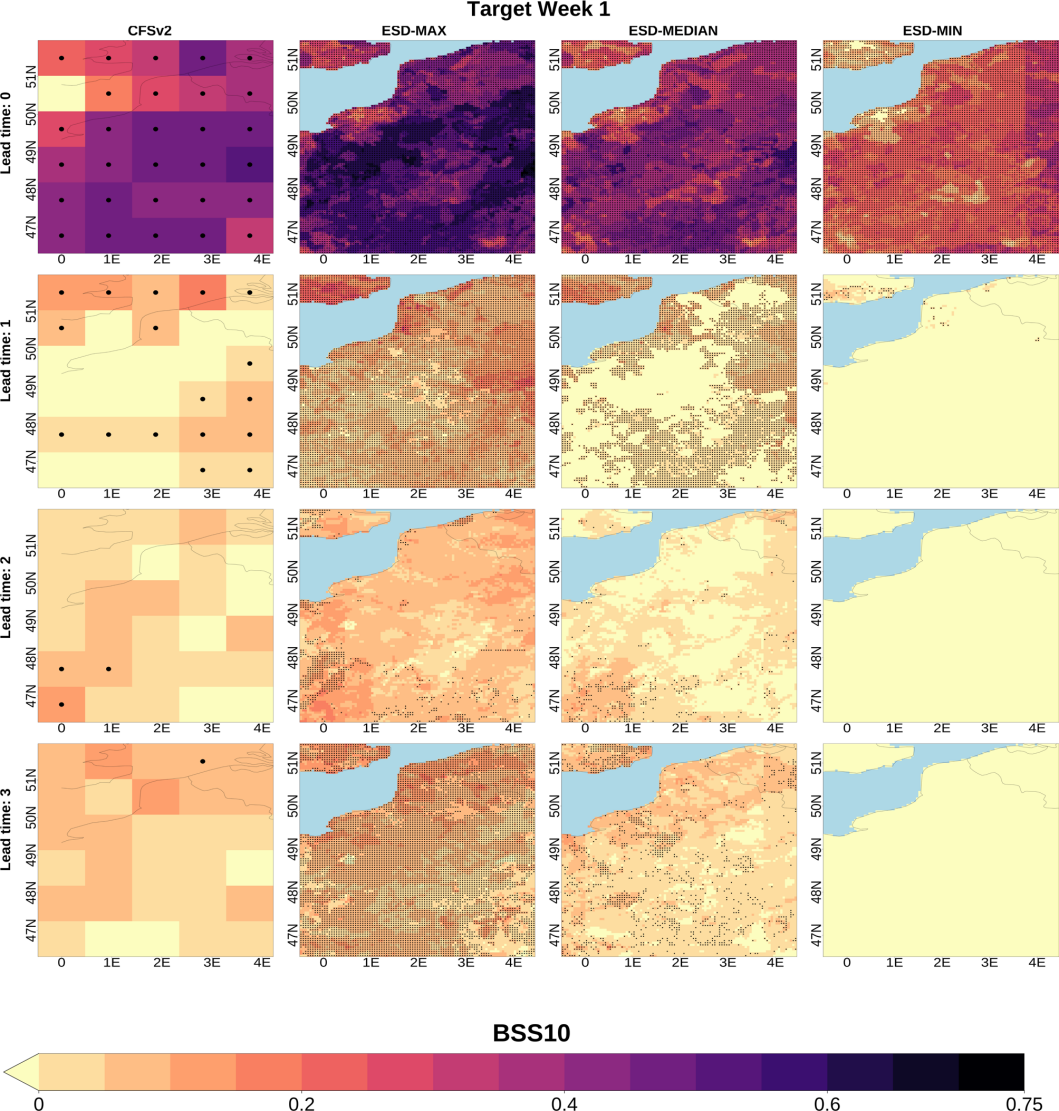
BSS10 of subseasonal temperature predictions for Target Week 1. BSS10 is computed over the 1999–2018 period. The first column displays the spatial distribution of the BSS10 calculated for the CFSv2 data in their native (coarse) grid while columns two to four respectively show the maximum, median and minimum skill values among the 27 tested downscaling methodologies for each grid point. BSS10 values at the grids with black dots are statistically significant at the 95% confidence level. The rows show the results for increasing lead times (from 1 to 4 weeks in advance). Negative skill values are shaded yellow. ESD: Empirical Statistical Downscaling.

We first compare the prediction skill of the downscaled ensemble subseasonal forecasts with that of CFSv2 at their native (coarse) resolution. Figure 2 illustrates the spatial distribution of the BSS10 metric calculated across 27 different tested methods for Target Week 1 (Target Week 2 and Target Week 3 are provided as Figs. S4 and S5, respectively, in the Supplementary Material). As seen in the panels within the first column, CFSv2 predictions in their native grid demonstrates higher skill for low temperature extremes than the climatological forecast across most grids at all lead times. The distribution of the median skill values for the downscaled methods (third column) closely aligns with that in the first column, indicating that, on average, these methods can effectively transfer the CFSv2 capabilities to the higher resolution. However, the comparison of the maximum and minimum BSS10 values (second and fourth columns, respectively) shows that this performance is not uniform among methods, with some methods struggling to maintain the skill at the high-resolution, particularly after lead time 1, while others exhibit statistically significant skill even in cases where CFSv2 does not (especially at lead time 3), and often surpass both the raw model and climatology in terms of BSS10. This analysis underscores the critical role of method selection in statistical downscaling.

**Fig. 3:**
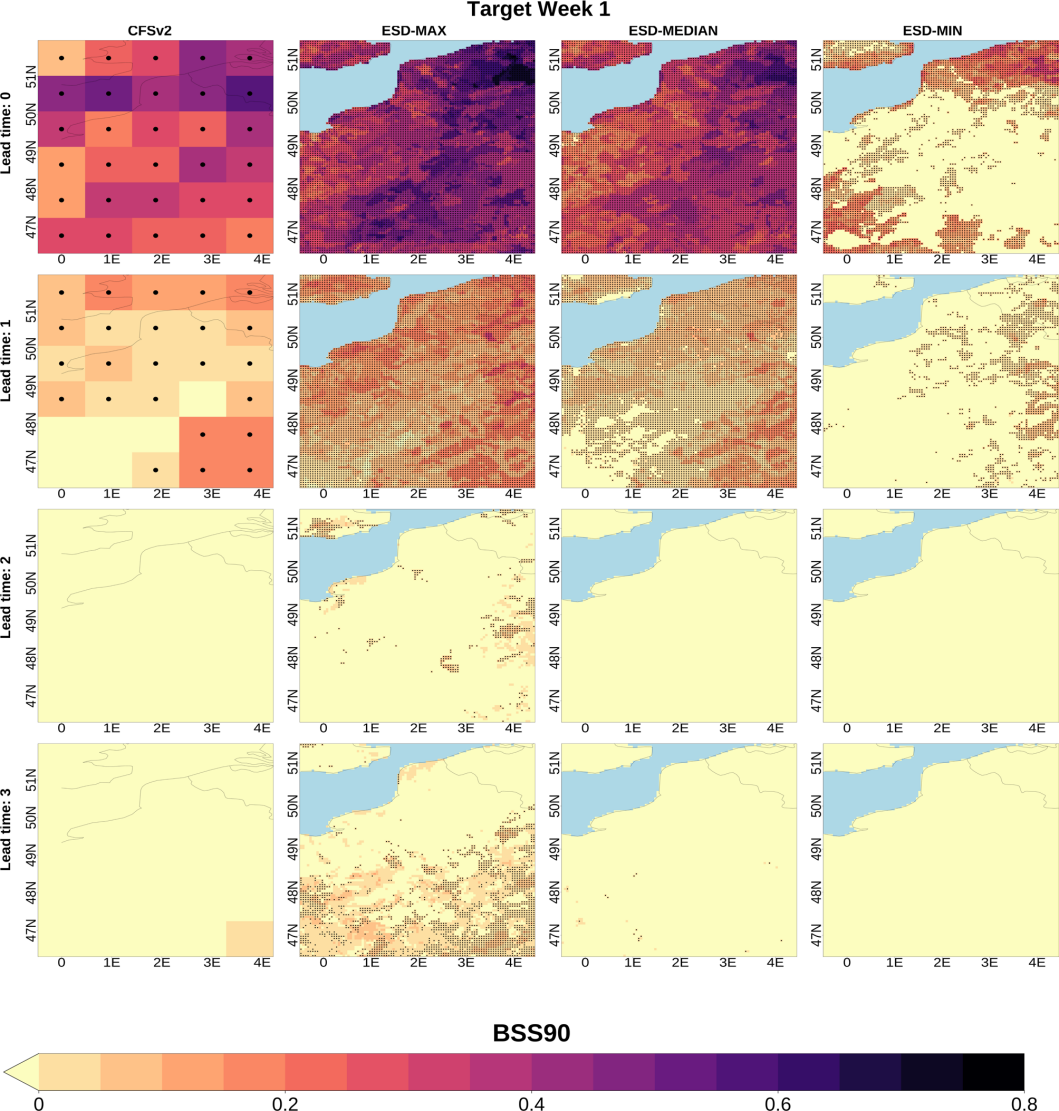
BSS90 of subseasonal temperature predictions for Target Week 1. BSS90 is computed over the 1999–2018 period. The first column displays the spatial distribution of the BSS90 calculated for the CFSv2 data in their native (coarse) grid while columns two to four respectively show the maximum, median and minimum skill values among the 27 tested downscaling methodologies for each grid point. BSS90 values at the grids with black dots are statistically significant at the 95% confidence level. The rows show the results for increasing lead times (from 1 to 4 weeks in advance). Negative skill values are shaded yellow. ESD: Empirical Statistical Downscaling.

For comparison, Fig. 3 presents the same information as displayed in Fig. 2 for BSS90 (high temperature extremes). Overall, the behavior of skill transfer through the downscaling methods generally parallels that of BSS10, though the magnitudes vary as CFSv2 exhibits systematically lower skill for BSS90 than for BSS10 for the target weeks in this study, reflecting higher skill in the lower (10th percentile) tail than in the upper (90th percentile) tail of weekly mean temperature anomalies. For instance, whereas CFSv2 shows skill at all lead times for BSS10, it demonstrates minimal skill after lead time 1 for BSS90. Consequently, the low skill in CFSv2 is passed through to the downscaled data, which, mostly, also lacks predictive capability. However, a notable exception to this outcome is observed for lead time 3 in the southern part of the study area (column 2, row 4) where some methods have skill at high resolution even when/where CFSv2 does not. A similar but weaker pattern is also observed in some grids for lead time 2. In general, the performance of the GCM and accordingly downscaling methods for the lead time 3 is better than that of the lead time 2. This might be linked to the emergence of predictability from slow components (e.g., soil-moisture/SST states and large-scale circulation modes), which become more influential by weeks 3–4 as the impact of initial atmospheric conditions weakens^15,75^. The outcomes stated above suggest that while substantial improvements on individual metrics are not generally expected, specific downscaling choices can enhance GCM skill locally and case dependently^36,76,77^.

### Which methods enhance the skill for high-end extremes and what are the underlying reasons for the enhancement?

**Fig. 4:**
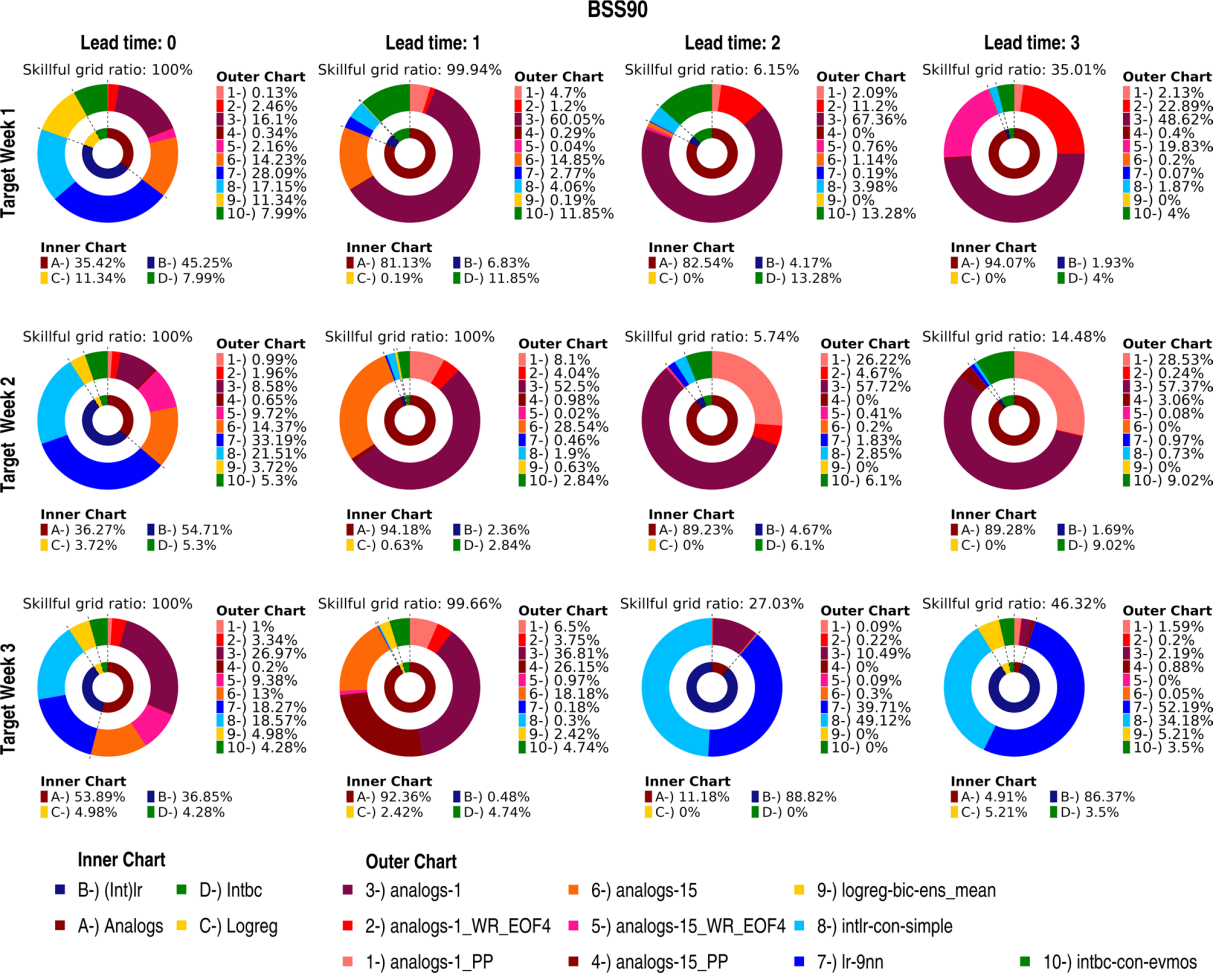
Donut charts illustrating the percentage of grid points where the methods perform best across target weeks and lead times for BSS90. The ratios are calculated based on skillful grid points only (i.e., BSS90 > 0). The skillful grid ratio (i.e., number of skillful grid points/total number of grid points) for each case is also shown above each subfigure. The inner chart displays the percentage of skillful grids where each of the four groups of statistical downscaling methods performed best, while the outer chart presents the same information for each of the ten best-performing methods.

This section discusses the methods that achieve improvements in the GCM skill as well as the potential reasons for their effectiveness. The discussion primarily focuses on the BSS90 metric to highlight the methods’ ability to predict extreme high-temperature events like heatwaves. However, to demonstrate that the best method may vary depending on the selected metric, the distributions of the best methods according to the BSS10 metric are shown in Fig. S6. Considering that it may be challenging to present all 27 methods, the ten most skillful methods (i.e. one or two of the most successful methods from each downscaling method group (Table S3); are shown in Fig. S6, Fig. 4 and Table S4. Our results indicate that the analogs method using the 5 and 15 best analogs are similar to those obtained using 1 analog (Fig. S7). Therefore, except for Fig. 4, only the results for the analogs-1 approaches are included in the following figures for better readability.

Figure 4 presents the percentages of method superiority for the BSS90 metric in the study area for the different lead times across the different target weeks. Table S4 complements this by summarising when each of these methods also was in the top-3 performing methods. To further understand these results, we first analyse in greater depth the scenario discussed in the previous section (Target Week 1, lead time 3, where the downscaled data appears more skillful than the CFSv2 data). Here, the downscaled forecast is more skillful than the climatological forecast in almost 35% of the grid points (Fig. 4). Among these, approximately 94% of the skillful grids identifies the analogs approaches as the most successful (see the inner chart in Fig. 4 for this specific case), and the Analogs-1, Analogs-1-WR_EOF4 and Analogs-15-WR_EOF4 methods together stand out as the best performers for approximately 91% of skillful grid points in total (outer chart) and 32% of all grid points (Table S4).

**Fig. 5:**
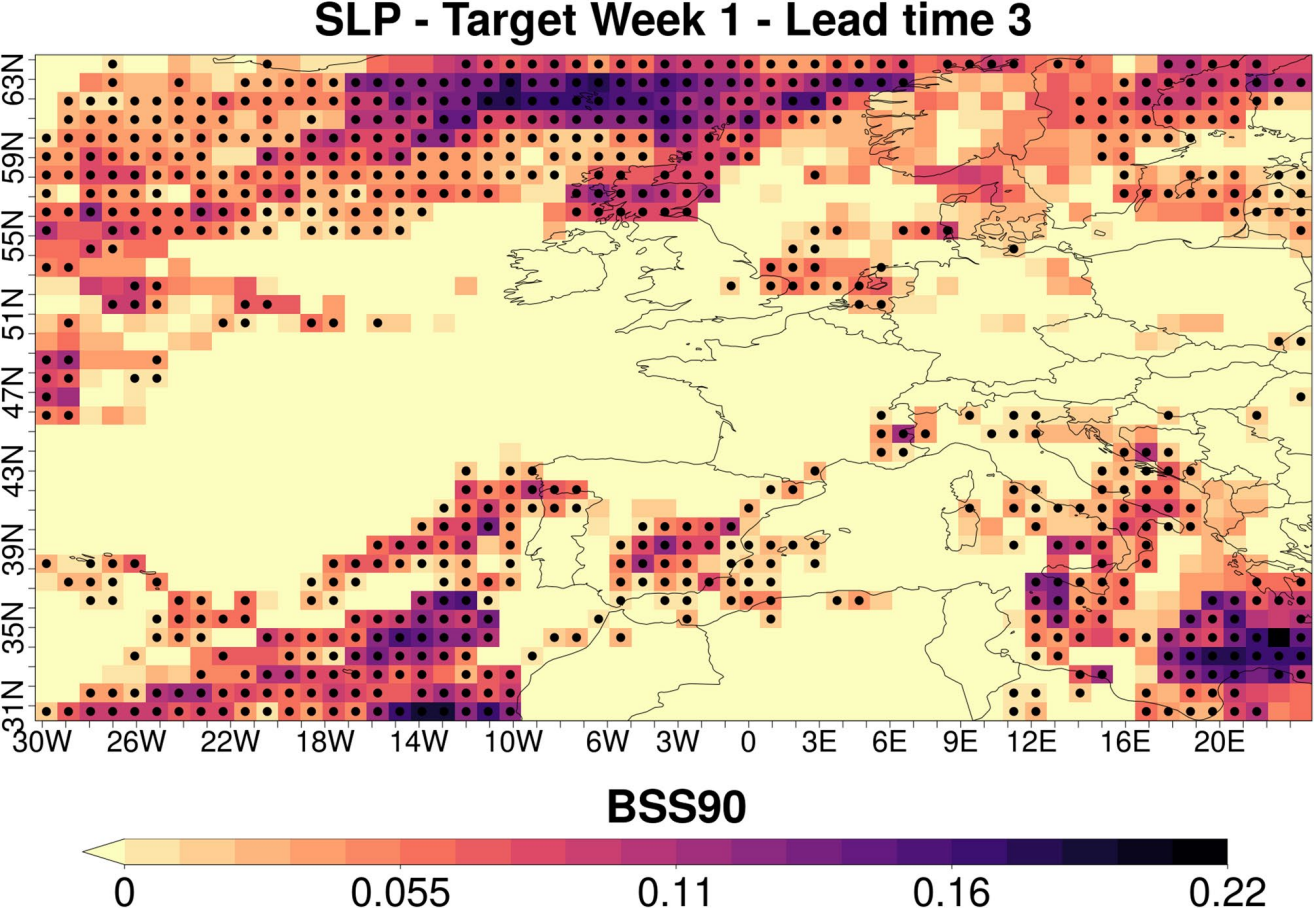
BSS90 for CFSv2-SLP over the North Atlantic region under Target Week 1 and lead time 3 conditions. ERA5 data is upscaled to the CFSv2 resolution before the skill calculation. BSS90 values at the grids with black dots are statistically significant at the 95% confidence level.

Two explanations have been suggested for downscaled predictions with greater skill than that of the coarse predictions they are based on. First, local information from the observed data can correct biases or low correlations that exist in the GCM predictions^78,79^. Second, skill from the large-scale GCM variables such as SLP can be transferred to the downscaled local-scale variables^29,80,81^. Since large-scale variables are not used as predictors in the MOS approach, skill enhancement in MOS may be attributed to the first reason. However, for PP and hybrid approaches the aim is to transfer the large-scale capabilities to the local scale. So, to understand the source of the additional skill provided by the hybrid methods for the Target Week 1 - lead time 3 scenario, we presented BSS90 for CFSv2-SLP over the large study domain (Fig. 5).

Moreover, as shown in Table S5, WR2 and WR3 are the dominant modes for Target Week 1, when extremely high temperature values occur across the region. Figure S3 illustrates that WR2 is characterized by low pressure and WR3 by high pressure over the northern or northeastern parts of the North Atlantic. Figure 5 further shows that, for Target Week 1 at lead time 3, the BSS90 of CFSv2-SLP is statistically significant over these northern North Atlantic regions, which correspond to the core pressure centres of the dominant WRs associated with local high temperatures over Paris during this target period. This information verifies that skillful SLP prediction by CFSv2 in regions influenced by the dominant WR can enhance downscaled temperature forecasts by transferring skill from large to local scales, even when the highest predictor skill occurs outside the target region. However, as noted by Goutham et al. (2023)^34^, subseasonal models often struggle to capture the onset and persistence of WRs like atmospheric blocking. This limits the effectiveness of WR-based approaches and explains why their added value is not consistent across all weeks and lead times. Correspondingly, when the dominant WR is not well predicted, simpler methods such as Analogs-MOS may yield better results.

### Spatial distributions of top-performing downscaling methods across lead times

**Fig. 6:**
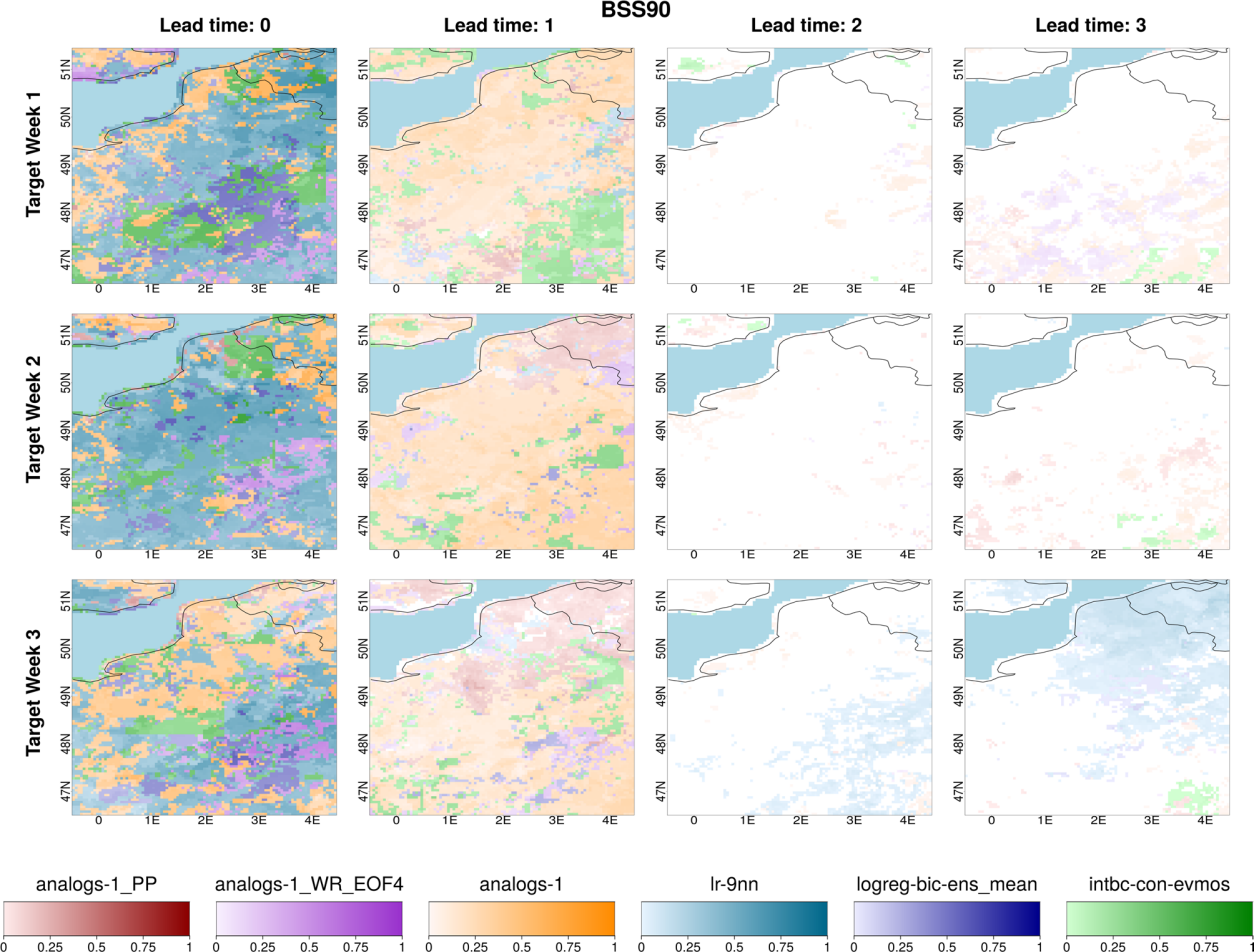
Spatial distribution showing which of the selected methods yields the highest BSS90 skill score for lead times 0–3 (columns) and Target Weeks 1–3 (rows). The selected methods include the best-performing approaches from each method group (three from analogs and one from each of linear regression, logistic regression, and bias correction). Different methods are represented by different colours, with shading intensity ranging from full capacity (BSS90 = 1) to white (BSS90 ≤ 0). Methods: analogs-1_PP (daily PP analogs, 1 analog); analogs-1_WR_EOF4 (daily hybrid analogs with 4 WRs, EOF-filtered); analogs-1 (weekly analogs, 1 analog); lr-9nn (weekly 9nn linear regression); logreg-bic-ens_mean (weekly logistic regression with bicubic interpolation and ensemble mean predictor); intbc-con-evmos (weekly evmos with conservative interpolation).

When examining the spatial distributions of the top-performing methods based on the BSS90 metric, consistent patterns emerge within lead times 0 and 1, though they are themselves different (Fig. 6). For lead time 0, lr-9nn is the best-performing method across the region among the six presented top methods, while analogs-1 is the best-performing method over the region for lead time (1) Specifically, lr-9nn shows strong performance in coastal and northeast areas for Target Week (2) Given that 9nn scans neighboring grids, this incorporation of the influence of sea grids on land grids may explain the enhancement compared to other models. This may also be linked to the topography as the flat terrain in coastal areas may facilitate the propagation of the information from neighboring grids to the target grid. This could explain the reappearance of skillful forecasts for the coastal and relatively flat area when using linear regression methods in lead time 3 for Target Week (3) At lead time 1, while the lr-9nn method continues to perform well (especially for Target Week 1 where the 9nn remained in the top 3 methods in 27% of grids), they are outperformed by the analog methods implemented with PP and hybrid approaches. This behavior can be attributed to the relatively improved performance of analog methods, rather than a decrease in the performance of regression models. For example, as shown in Table S4, for Target Week 3, the linear regression methods (i.e., lr-9nn and int-con-simple) are the best-performing methods among the selected 10 methods in 37% of the grids at lead time 0, but in less than 4% of the grids at lead time 1. Nevertheless, they remain among the top three methods in 66% and 53% of the grids, respectively, at lead time 1. Conversely, the analogs-15-PP method is the best method in less than 2% of the grids at lead time 0, but becomes the best method in 26% of the grids at lead time 1 and is among the top 3 methods in 40% of the grids. Besides, particularly noteworthy is the success of the analogs method integrated with the hybrid approach in the southern region during Target Week 1 at lead time 3. The increased success of methods using hybrid and PP approaches with longer lead times is associated with the large-scale variable used as a predictor (i.e. SLP over the North Atlantic). This variable not only has higher skill at longer lead times compared to temperature itself, but it also influences temperature with a lag, rather than having an immediate effect^82^.

### Comparison of method skill from daily versus weekly data in subseasonal downscaling

**Fig. 7:**
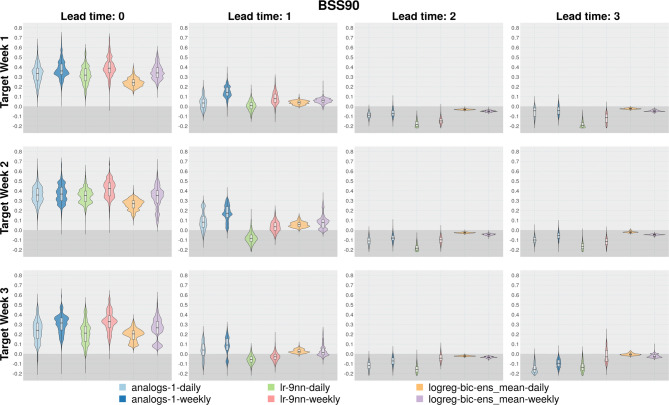
Distributions of BSS90 skill scores for models established using daily and weekly data for analog, linear- and logistic-regression models. The boxplots encompass BSS90 values across all grids in the study domain. Areas with negative skill values are shaded with a dark gray background. analogs-1-daily: Analogs model built with daily data, selecting 1 analog and MOS approach. analogs-1-weekly: Analogs model built with weekly data, selecting 1 analog and MOS approach. lr-9nn-daily: 9nn linear regression model built with daily data. lr-9nn-weekly: 9nn linear regression model built with weekly data. logreg-bic-ens_mean-daily: Bicubic interpolation plus logistic regression model using ensemble mean value as the predictor, built with daily data. logreg-bic-ens_mean-weekly: Bicubic interpolation plus logistic regression model using ensemble mean value as the predictor, built with weekly data.

All methods except those that use bias correction were tested to investigate whether using daily data enhances the skill of the downscaled subseasonal predictions compared to the models constructed with weekly data. Even though daily data is initially expected to represent extremes better due to its wider spread compared to weekly data (Fig. S8), downscaling with weekly data generally tends to be more skillful than daily data across all methods (Fig. 7). One reason for this outcome is that, even when models are built with daily data, skill evaluations are conducted on weekly averages. While establishing models with longer datasets is undoubtedly important, increasing the data length at the temporal scale of skill evaluation, through approaches such as extended windows, appears to be particularly crucial. Another possible reason may arise from the construction of most methods using MOS. It is highly likely that GCM models, which already have difficulty predicting accurate local-scale variables such as temperature, struggle with increased uncertainty at higher temporal scales, and thus are less able to provide accurate predictions which are then passed through to the downscaled data. As summarized above, incorporating large-scale variables and WRs into models developed at a daily scale can still enhance model performance and has the potential to further improve it. However, our results reveal that a simple analogs method with weekly data and the MOS approach frequently yield the most accurate results. Therefore, before applying complex subseasonal downscaling algorithms, it is essential to thoroughly evaluate in which cases PP or hybrid approaches are justifiable, as well as how and to what extent they can enhance skill. This careful consideration will help maintain the study’s simplicity while avoiding excessively high computational costs.

## Summary and conclusions

This study evaluated the potential of statistical downscaling to transfer subseasonal temperature-forecast skill from coarse (~ 100 km) to high (~ 5 km) resolution. We conducted a comparative assessment of 27 methods. The results provide insights into how large-scale predictors, workflows, and techniques shape weekly-scale forecast quality, with implications for extreme temperature planning. We used CFSv2 as the coarse-resolution subseasonal prediction for downscaling and CERRA as the high-resolution reference. We analysed the performance of four downscaling approaches (bias correction, linear regression, logistic regression, and analogs) under different set-ups. To understand how far in advance it is possible to provide high-resolution information that is more skillful than climatology, we tested downscaling processes for each CFSv2 model initiated one, two, three and four weeks before the three target weeks of the Paris 2024 Olympics. We thus provide the following conclusions and suggestions for possible future work:

- The best-performing downscaling method varies depending on the lead time, target week, and the chosen skill metric. For instance, the analogs family shows the highest skill in Target Weeks 1–2 when considering both skill metrics (BSS10 and BSS90). By contrast, in Target Week 3 linear regression methods achieve the best performance, particularly the nine nearest neighbors multiple linear regression method (i.e., lr-9nn), while analogs remain among the top-performing approaches. These differences may be due to the varying climatic conditions of each target week or the state of the hydroclimatological situations when the CFSv2 model is initialized. This case dependence prevents us from claiming a universally best method; instead, these approaches are best viewed as promising candidates for future studies and broader evaluations.
- The superior performance of the analogs and lr-9nn methods over other approaches may stem from their ability to capture both spatial and temporal large-scale patterns. Unlike other methods that rely solely on overlapping grids, these non-local approaches scan the entire or surrounding domain, a characteristic that likely contributes to their enhanced skill. These results are relevant for the implementation of downscaling approaches in operational climate services providing subseasonal predictions for sector-specific decision-making, including heat-risk management, energy demand planning, agriculture, and emergency preparedness such as wildfire and flood risk management.
- On average, the median value of the methods successfully transfers subseasonal forecast information to high resolution and maintains the CFSv2 skill, but within the different methods there is considerable variability with some techniques significantly degrading the CFSv2 skill while some even manage to enhance it. PP-oriented methods leverage the strengths of both large-scale variables and observations to increase CFSv2 skill, while MOS models primarily benefit from observational data. However, since the evmos techniques used in this study adjust only certain parameters of the underlying distribution (i.e., the mean and variance in our case) without establishing pairwise alignment between input and output, no additional skill improvement is expected for metrics that are insensitive to the mean, such as BSS10 and BSS90. The only difference in skill values between the downscaled data via evmos techniques and CFSv2 arises from the application of interpolation and cross-validation during the downscaling process.
- Hybrid approaches incorporating WRs may perform well when the dominant North Atlantic WRs associated with local events are skillfully predicted by the GCM, especially if their key drivers (e.g., SLP) exhibit statistically significant skill over the relevant circulation domains. In such situations, including WR information in the downscaling framework can help improve the representation of high-temperature extremes. Conversely, when the models show limited skill at predicting the large-scale drivers, simpler approaches constructed with weekly data may be preferable, as they can provide competitive skill with lower methodological complexity and computational cost.
- Downscaled forecasts at lead time 3 tend to be more skillful than at lead time 2, likely because by this range the model begins to capture predictive skill from slow-varying components and large-scale circulation, while lead time 2 represents a transitional stage where the influence of initial atmospheric conditions has faded but slower drivers have not yet fully emerged. Given our limited sample, this lead-time behavior should be interpreted as case-specific, and its generality should be evaluated in future studies with larger samples and carefully designed setups.
- The models constructed with weekly data outperform those constructed with daily data at the subseasonal scale. This suggests that, rather than using a dataset with higher temporal resolution to extend the length of the data in model construction, it is more logical to use approaches such as extended windows, which preserve the weekly scale while increasing the data number, as demonstrated in this study. Nonetheless, daily data can still be useful for PP approaches where day-by-day correspondence is the focus.
- While this study assesses the added value of downscaling relative to the raw GCM forecasts rather than maximizing absolute skill, further studies are required to identify additional large-scale predictors and modelling strategies that could improve the operational usefulness and skill of subseasonal downscaling. Here, we demonstrated the benefits of incorporating large-scale SLP fields, which can help capture drivers relevant for local-scale temperature variability. Future work could extend this framework by testing additional predictors (e.g., upper-air temperature, wind, and geopotential height, as well as land-surface states such as soil moisture), increasing effective ensemble information (e.g., larger ensembles or driver-informed subsampling), and using higher-resolution observational references. Importantly, the methodology presented here can also be applied to multi-model subseasonal systems, and complemented by AI-based downscaling approaches alongside the statistical methods tested in this study, to leverage potentially more skillful operational settings. Finally, although we focused on temperature, the same principles could be adapted to multivariate heat-related indices such as Universal Thermal Climate Index (UTCI), provided that the relevant drivers are carefully considered and the workflow is tailored accordingly.

Lastly, we emphasize that the primary aim here is to provide a methodological assessment of whether statistical downscaling can preserve or enhance extreme-event skill at the subseasonal weekly scale under different conditions, rather than to deliver a comprehensive evaluation of subseasonal predictability across seasons or regions. Our findings suggest that caution is required when choosing a method rather than recommending any in particular. The selected target weeks offer an operationally relevant testbed, but the predictability behavior and method ranking are inevitably conditioned on the meteorological characteristics of these specific periods. Accordingly, while the results provide methodological insight into downscaling behavior under a consistent setup, they should be interpreted as case-specific, and extending the framework to a larger number of cases is a key next step to assess robustness and generality.

## Supplementary Information

Below is the link to the electronic supplementary material.

Supplementary Material 1

## Data Availability

CFSv2 sub-seasonal forecasts are available on the NCEP NOMADS server, while CERRA, E-OBS and ERA5 data can be accessed through the Copernicus Climate Data Store.
