# Supplementary material for: Assessing the utility of statistical downscaling for subseasonal temperature forecasts
Source: Sci Rep. 2026 Mar 26;16:15127. doi: 10.1038/s41598-026-45067-2 (PMC13172455; doi:10.1038/s41598-026-45067-2)
Supplement: Supplementary file 1 — Supplementary Material 1 [file 41598_2026_45067_MOESM1_ESM.docx]

**Supplementary Figures, Tables, and Analyses**

Eren Duzenli^1^, Jaume Ramon^1^, Verónica Torralba^1^, Sam Pickard^1^, Ángel G Muñoz^1^, Dragana Bojovic^1^

^1^Earth Sciences Department, Barcelona Supercomputing Center (BSC), Barcelona, Spain

Corresponding author: Eren Duzenli ([eren.duzenli@bsc.es](mailto:eren.duzenli@bsc.es))

This file contains eight supplementary figures, five supplementary tables, and additional analyses related to observational data uncertainty and quality.

ASSESSING THE CERRA REANALYSIS USING E-OBS AS A REFERENCE

A reanalysis dataset was used as the reference due to the lack of spatiotemporally continuous, high-resolution observational data for the study area. The highest spatial resolution observational dataset available in the region was the E-OBS gridded dataset[^1^](https://www.zotero.org/google-docs/?qHqd8p), with an approximate resolution of 10 km. Compared to E-OBS, CERRA offers a key advantage with its higher resolution (approximately 5 km). Still, to assess the representativeness of CERRA for observational data, a correlation analysis was conducted between CERRA and E-OBS. Spearman’s rank correlation coefficients were calculated from the daily data of the 20-year datasets for each overlapping grid between the products, separately for each of the three weeks of the Paris 2024 Olympics. Before performing the analysis, CERRA data were interpolated to the E-OBS resolution using conservative interpolation. The corresponding results are presented in Figure S1 and Table S1. Figure S1 displays a scatter plot of 20 years of daily values for CERRA and E-OBS at 10 km grids, while Table S1 summarizes the quantile values of the distribution of the correlation values calculated at the grids. The findings reveal that daily correlation values exceed 0.93 in more than 90% of the grids for all target weeks. Thus, CERRA not only provides an opportunity of downscaling coarse-resolution subseasonal predictions to a higher-resolution field (i.e. 5 km) but also demonstrates strong agreement with the observational data.


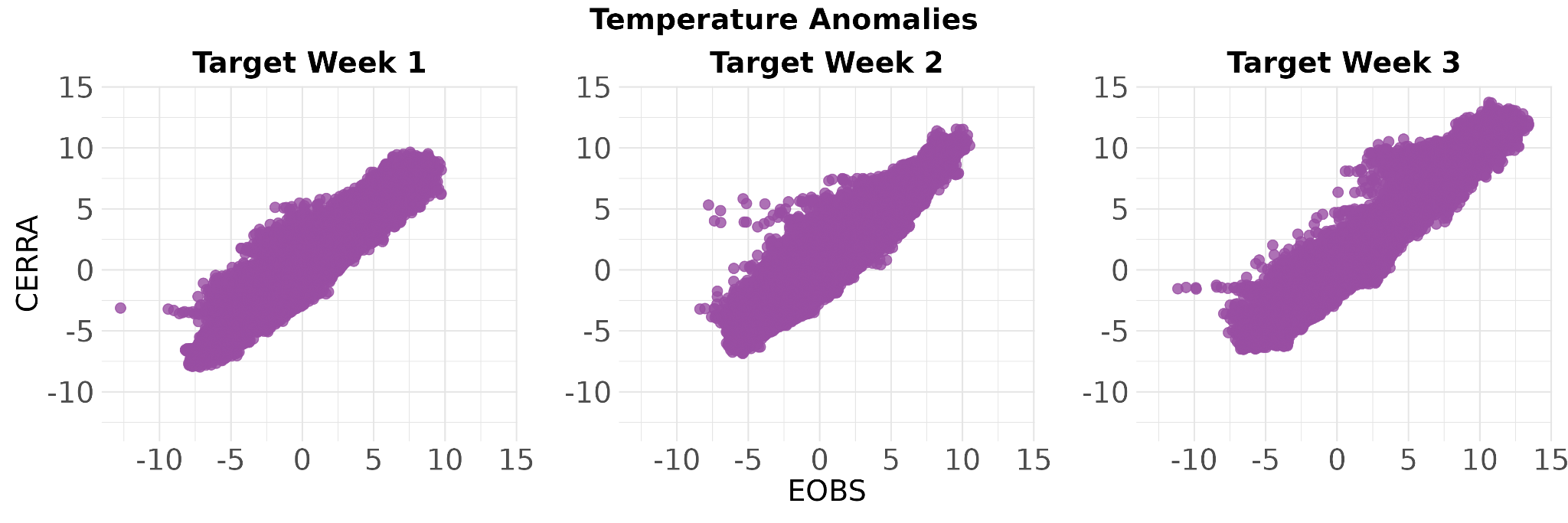
Figure S1 Scatter plot of the daily mean temperature anomalies between the CERRA and E-OBS products, including the data from all grids within the study domain.

Table S1 Quantile values of the distribution of correlations calculated between the CERRA and E-OBS datasets for selected percentiles. The correlation values are derived from the daily data of the 20-year datasets, calculated separately for each overlapping grid.

|  | 0% | 10% | 25% | 50% | 75% | 90% | 100% |
| --- | --- | --- | --- | --- | --- | --- | --- |
| Target Week 1 | 0.58 | 0.93 | 0.94 | 0.95 | 0.96 | 0.97 | 0.99 |
| Target  Week 2 | 0.59 | 0.93 | 0.95 | 0.96 | 0.97 | 0.97 | 0.99 |
| Target  Week 3 | 0.78 | 0.93 | 0.94 | 0.96 | 0.96 | 0.97 | 0.98 |

Moreover, Table S2 presents the minimum, mean, and maximum values of the spatially averaged daily mean temperatures over a 20-year study period for the CERRA and E-OBS products, calculated for the target weeks across the study domain. The minimum and mean daily mean temperature values for the target weeks are generally quite similar. However, in terms of maximum daily mean temperature, Target Week 3 reached higher values than the other weeks. Additionally, Figure S2 displays the daily mean temperature values of the CERRA product by year and target week, showing spatial averages over the study area. Analyzing the years in which temperatures fell within the upper 10% reveals that, in some years, more than five days during the target weeks exceeded this threshold. For instance, during the 2003 heatwave, temperatures in Target Week 3 remained in the upper 10% for seven consecutive days. This situation also highlights the utility of the weekly analyses (i.e. subseasonal scale) for the understanding and characterization of heatwave episodes.

Table S2 The minimum, mean, and maximum values of the spatially averaged daily mean temperatures during the study period (1999-2018) by target week and observational dataset. Temperature values are provided in degrees Celsius.

|  |  | **Min** | **Mean** | **Max** |
| --- | --- | --- | --- | --- |
| Target  Week 1 | CERRA | 16.10 | 19.19 | 23.21 |
|  | E-OBS | 16.63 | 19.73 | 24.16 |
| Target  Week 2 | CERRA | 17.25 | 19.16 | 22.53 |
|  | E-OBS | 17.26 | 19.59 | 23.02 |
| Target  Week 3 | CERRA | 16.19 | 18.6 | 26.63 |
|  | E-OBS | 16.65 | 19.03 | 27.45 |

EVALUATION OF HISTORICAL TEMPERATURE DATA OVER THE STUDY AREA


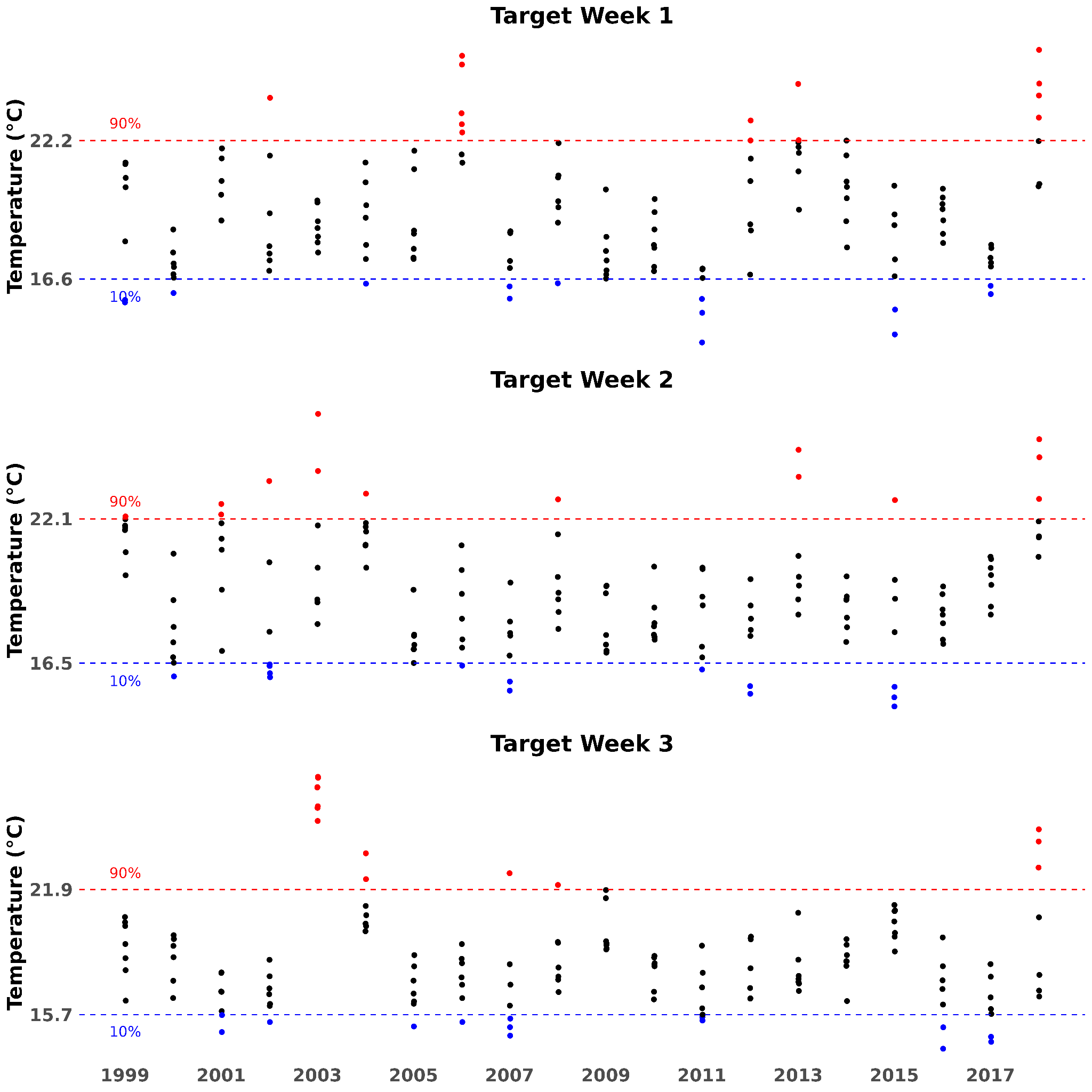
Figure S2 Daily mean temperature of the CERRA product observed during the target weeks by year. In the figure, the 90th percentile represents the lower limit of the upper 10%, while the 10th percentile indicates the upper limit of the lower 10%. The presented values are spatial averages.

THE MOST SUCCESSFUL METHODS

Table S3 The most successful downscaling methods from each downscaling method group. "nn" represents the number of selected analogs.

| **Method** | **Explanation** |
| --- | --- |
| analogs-nn | Analogs model built with weekly data, selecting nn (i.e. 1, 5 or 15) analog(s) |
| analogs-nn_WR_EOF4 | Hybrid analogs model using daily data, selecting nn (i.e. 1, 5 or 15) analog(s), and using 4 WRs obtained with EOF pre-filtering |
| analogs-nn_PP | Analogs model built with daily data, selecting nn (i.e. 1, 5 or 15) analog(s) and PP approach |
| intlr-con-simple | Conservative interpolation plus simple linear regression model built with weekly data |
| lr-9nn | 9nn linear regression model built with weekly data |
| logreg-bic-ens_mean | Bicubic interpolation plus logistic regression model using ensemble mean value as the predictor, built with weekly data |
| intbc-con-evmos | Conservative interpolation plus evmos model built with weekly data |

WEATHER REGIMES FOR THE TARGET WEEKS


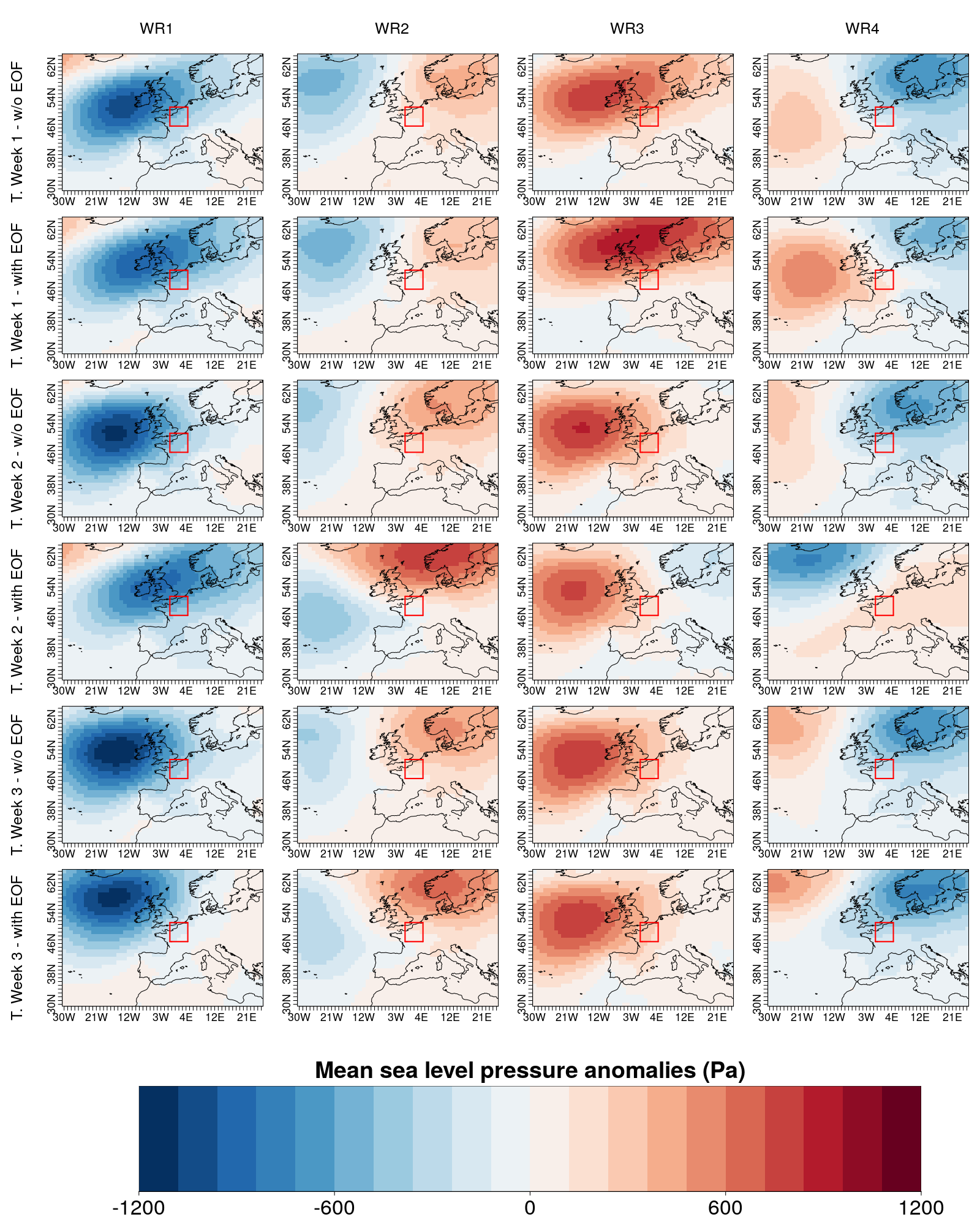


Figure S3 WRs obtained with and without EOF pre-filtering with respect to the target weeks of the Paris 2024 Olympics. "T. Week" on the figure refers to the "Target Week". The red square indicates the study domain (i.e., the Paris region).

PERFORMANCE OF THE MOST SUCCESSFUL METHODS

Table S4 The percentages of how often the methods are the best or one of the top three models among the ten selected methods, based on the BSS90 metric, across all grids. If the maximum skill value on a grid is negative, no method is considered the best for that grid.

|  | **Method** | **Lead time 0** | | **Lead time 1** | | **Lead time 2** | | **Lead time 3** | |
| --- | --- | --- | --- | --- | --- | --- | --- | --- | --- |
|  |  | The best (%) | Top three  (%) | The  best  (%) | Top three  (%) | The best  (%) | Top three  (%) | The best  (%) | Top three  (%) |
| Target Week 1 | analogs_1 | 16 | 43 | 60 | 89 | 4 | 5 | 17 | 26 |
|  | analogs_1-PP | <2 | <2 | 5 | 20 | <2 | 6 | <2 | 29 |
|  | analogs_1-WR_EOF4 | 3 | 10 | <2 | 11 | <2 | 6 | 8 | 28 |
|  | analogs_15 | 14 | 41 | 15 | 62 | <2 | <2 | <2 | <2 |
|  | analogs_15-PP | <2 | <2 | <2 | <2 | <2 | <2 | <2 | <2 |
|  | analogs_15-WR_EOF4 | 2 | 14 | <2 | 4 | <2 | <2 | 7 | 14 |
|  | lr-9nn | 28 | 75 | 3 | 27 | <2 | <2 | <2 | <2 |
|  | int-con-simple | 17 | 54 | 4 | 20 | <2 | <2 | <2 | <2 |
|  | logreg-bic-ens_mean | 11 | 25 | <2 | 7 | <2 | <2 | <2 | <2 |
|  | intbc-bic-evmos | 8 | 36 | 12 | 59 | <2 | <2 | <2 | 4 |
| Target  Week 2 | analogs_1 | 8 | 31 | 52 | 89 | 3 | 5 | 8 | 13 |
|  | analogs_1-PP | <2 | 2 | 8 | 19 | <2 | 6 | 4 | 14 |
|  | analogs_1-WR_EOF4 | 2 | 15 | 4 | 26 | <2 | 6 | <2 | 12 |
|  | analogs_15 | 14 | 40 | 28 | 76 | <2 | <2 | <2 | <2 |
|  | analogs_15-PP | <2 | 2 | <2 | 5 | <2 | <2 | <2 | <2 |
|  | analogs_15-WR_EOF4 | 10 | 23 | <2 | 4 | <2 | <2 | <2 | <2 |
|  | lr-9nn | 33 | 84 | <2 | 6 | <2 | <2 | <2 | <2 |
|  | int-con-simple | 22 | 60 | 2 | 12 | <2 | <2 | <2 | <2 |
|  | logreg-bic-ens_mean | 4 | 12 | <2 | 11 | <2 | <2 | <2 | <2 |
|  | intbc-bic-evmos | 5 | 32 | 3 | 52 | <2 | <2 | <2 | 3 |
| Target  Week 3 | analogs_1 | 27 | 50 | 36 | 66 | 3 | 4 | <2 | <2 |
|  | analogs_1-PP | <2 | 3 | 6 | 35 | <2 | 26 | <2 | 36 |
|  | analogs_1-WR_EOF4 | 3 | 15 | 4 | 31 | <2 | 14 | <2 | 14 |
|  | analogs_15 | 13 | 46 | 18 | 57 | <2 | <2 | <2 | <2 |
|  | analogs_15-PP | <2 | <2 | 26 | 40 | <2 | <2 | <2 | <2 |
|  | analogs_15-WR_EOF4 | 9 | 24 | <2 | 13 | <2 | <2 | <2 | <2 |
|  | lr-9nn | 18 | 66 | <2 | 3 | 11 | 17 | 24 | 38 |
|  | int-con-simple | 19 | 53 | <2 | 6 | 13 | 19 | 16 | 34 |
|  | logreg-bic-ens_mean | 5 | 13 | 3 | 10 | <2 | <2 | 2 | 13 |
|  | intbc-bic-evmos | 4 | 30 | 5 | 37 | <2 | <2 | <2 | <2 |

DOMINANT WEATHER REGIMES DURING THE WARMEST YEARS

Table S5 Percentages of days in the three warmest years assigned to different WRs. The three warmest years for Target Week 1 are 2006, 2013, and 2018; for Target Week 2, they are 1999, 2004, and 2018; and for Target Week 3, they are 2003, 2004, and 2018.

|  |  | **WR1** | **WR2** | **WR3** | **WR4** |
| --- | --- | --- | --- | --- | --- |
| Target  Week 1 | w/o EOF | 12.38 | 37.14 | 38.09 | 12.38 |
|  | with EOF | 12.38 | 36.19 | 31.43 | 20 |
| Target  Week 2 | w/o EOF | 27.61 | 32.38 | 20 | 20 |
|  | with EOF | 20.95 | 26.67 | 19.05 | 33.33 |
| Target  Week 3 | w/o EOF | 19.05 | 44.76 | 22.86 | 13.33 |
|  | with EOF | 29.52 | 33.33 | 22.86 | 14.29 |

SUPPLEMENTARY FIGURES REFERENCED IN THE RESULTS AND DISCUSSION


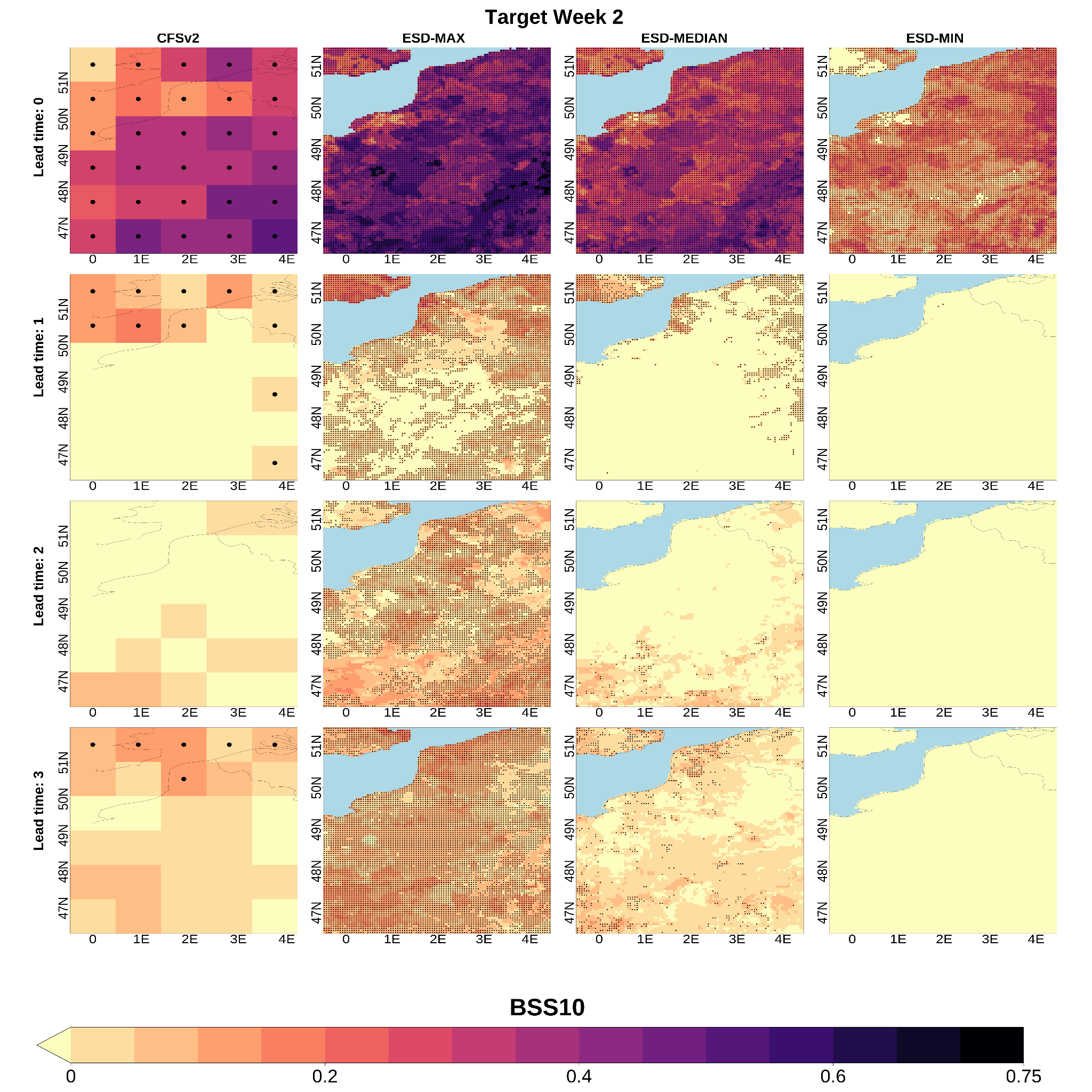
Figure S4 BSS10 of subseasonal temperature predictions for Target Week 2. BSS10 is computed over the 1999-2018 period. The first column displays the spatial distribution of the BSS10 calculated for the CFSv2 data in their native (coarse) grid while columns two to four respectively show the maximum, median and minimum skill values among the 27 tested downscaling methodologies for each grid point. BSS10 values at the grids with black dots are statistically significant at the 95% confidence level. The rows show the results for increasing lead times (from 1 to 4 weeks in advance). Negative skill values are shaded yellow. ESD: Empirical Statistical Downscaling.


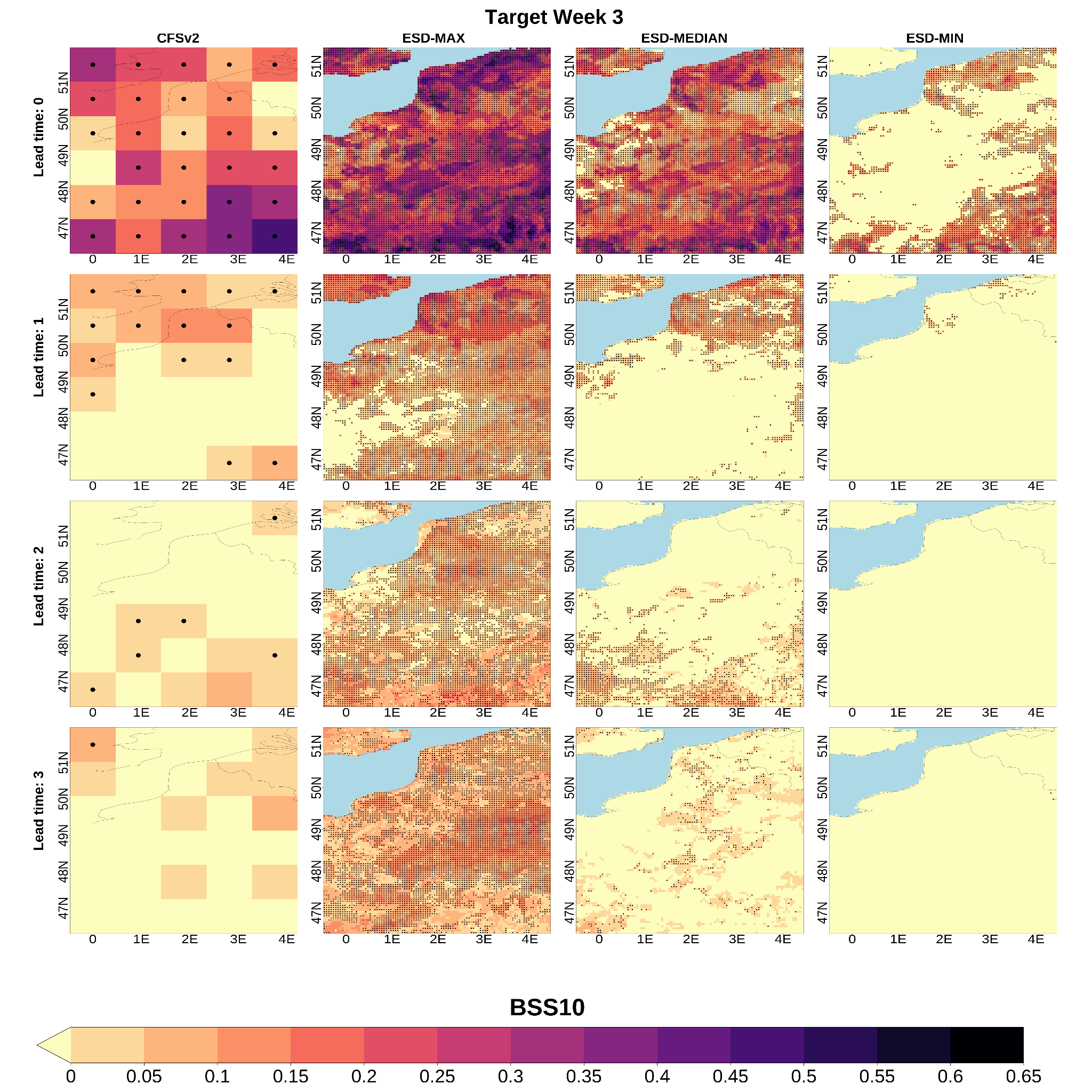
Figure S5 BSS10 of subseasonal temperature predictions for Target Week 3. BSS10 is computed over the 1999-2018 period. The first column displays the spatial distribution of the BSS10 calculated for the CFSv2 data in their native (coarse) grid while columns two to four respectively show the maximum, median and minimum skill values among the 27 tested downscaling methodologies for each grid point. BSS10 values at the grids with black dots are statistically significant at the 95% confidence level. The rows show the results for increasing lead times (from 1 to 4 weeks in advance). Negative skill values are shaded yellow. ESD: Empirical Statistical Downscaling.


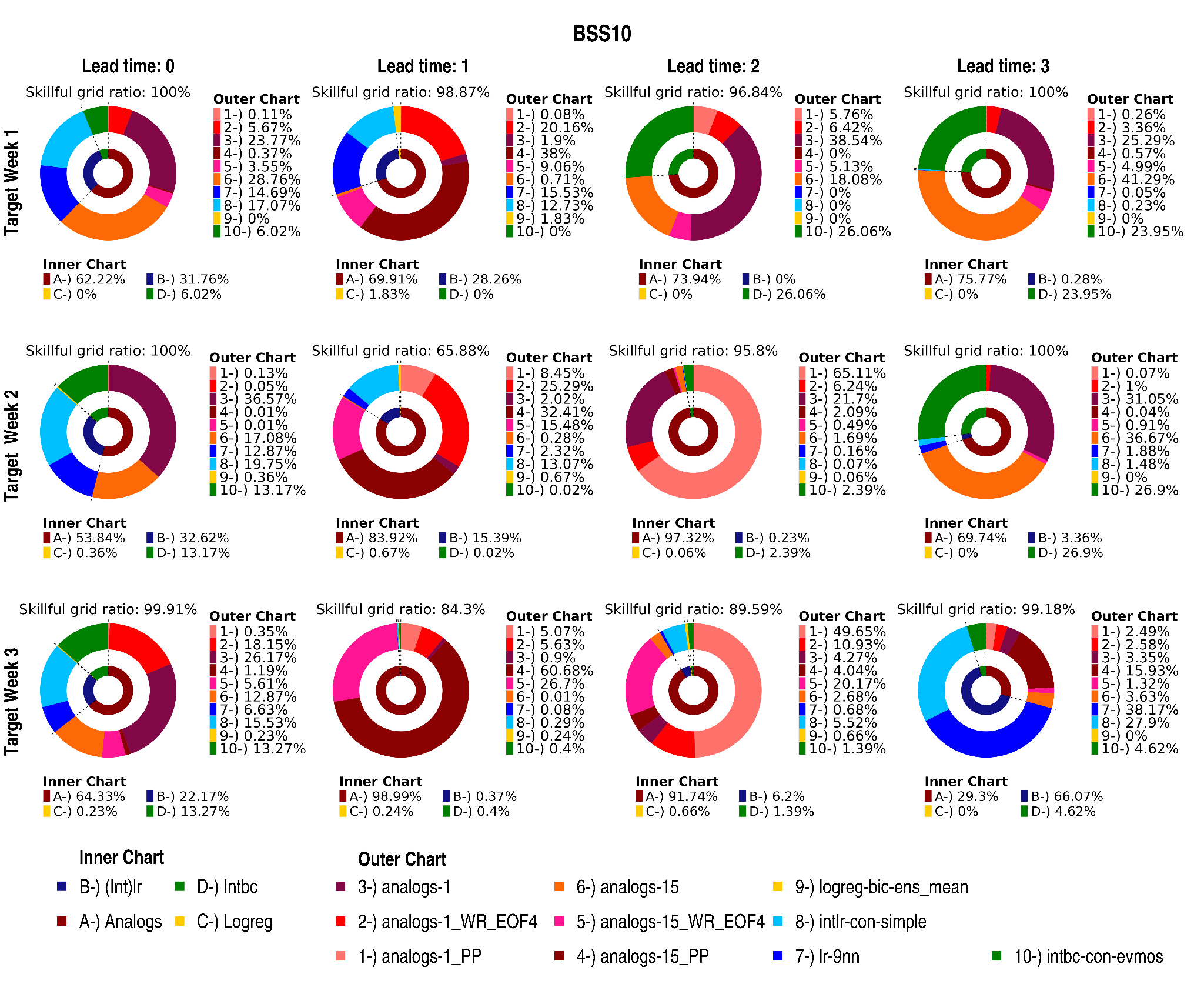
Figure S6 Donut charts illustrating the percentage of grid points where the methods perform best across target weeks and lead times for BSS10. The ratios are calculated based on skillful grid points only (i.e., BSS10 > 0). The skillful grid ratio (i.e., number of skillful grid points / total number of grid points) for each case is also shown above each subfigure. The **inner chart** displays the percentage of skillful grids where each of the four groups of statistical downscaling methods performed best, while the **outer chart** presents the same information for each of the ten best-performing methods.


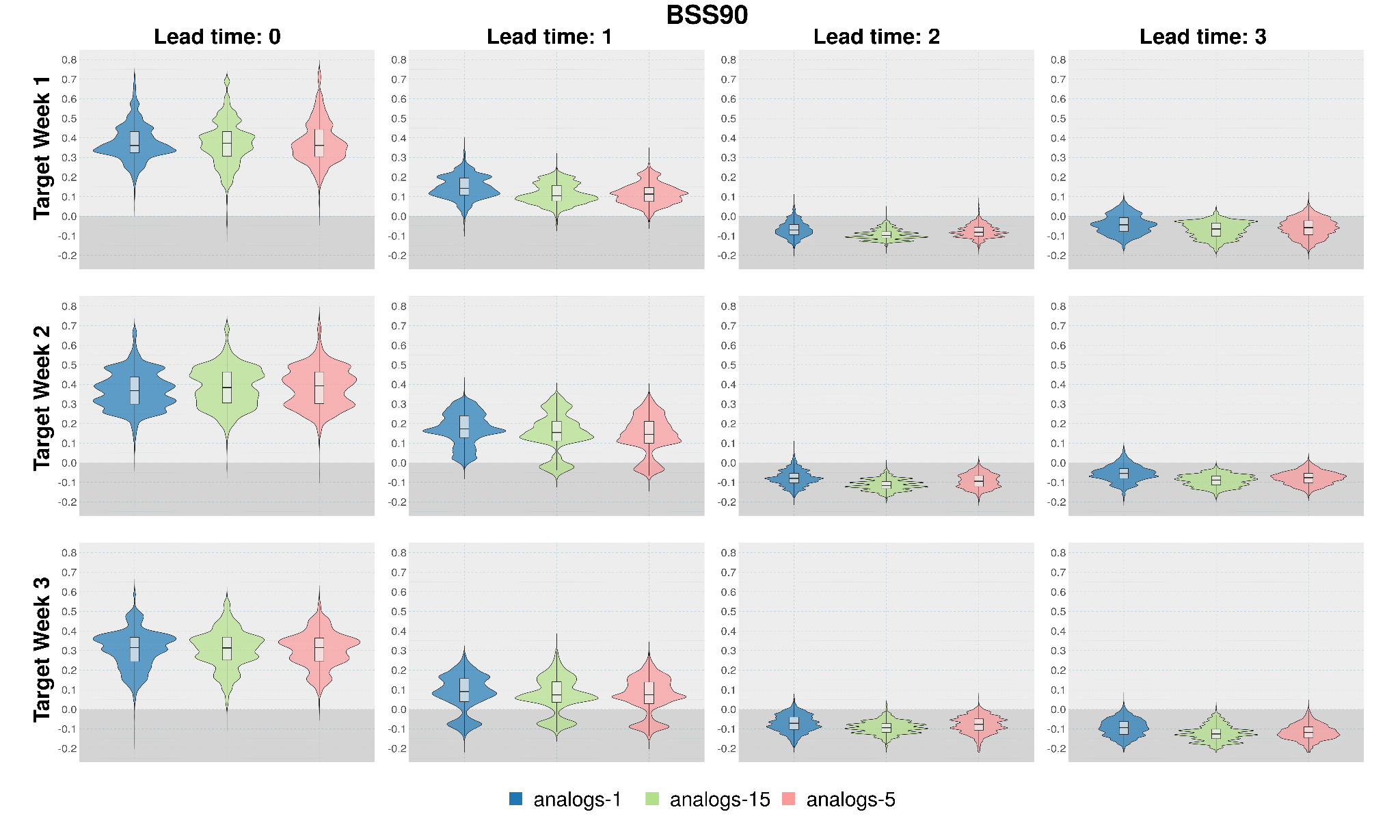
Figure S7 Distributions of BSS90 skill scores for the analogs models with 1, 5 and 15 analogs. The boxplots encompass BSS90 values across all grids in the study domain. Areas with negative skill values are shaded with a dark gray background.  **analogs-1**: Analogs model built with weekly data, selecting 1 analog and MOS approach. **analogs-5**: Analogs model built with weekly data, selecting 5 analogs and MOS approach. **analogs-15**: Analogs model built with weekly data, selecting 15 analogs and MOS approach.


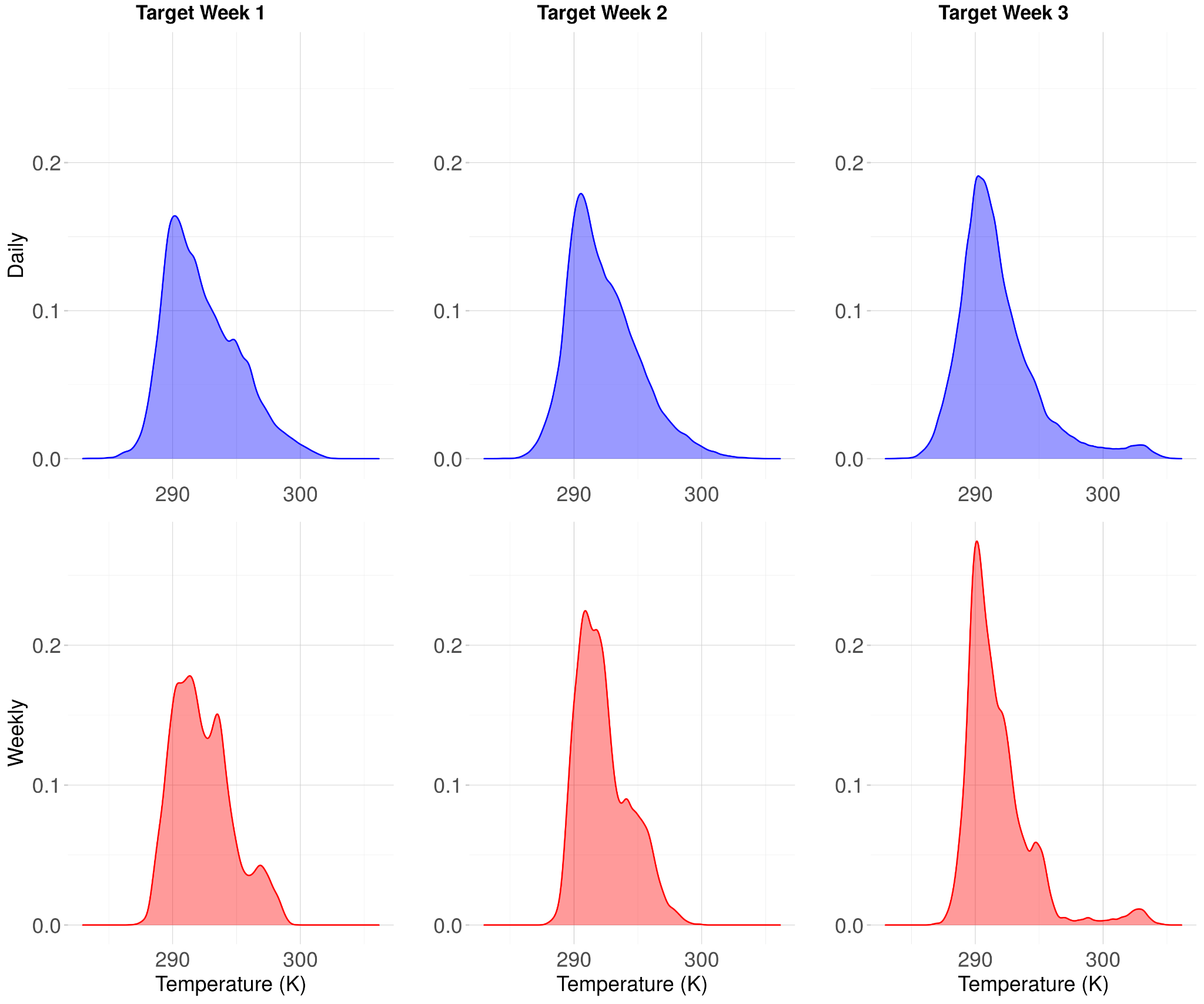


Figure S8 Probability density function for weekly and daily CERRA datasets. Mean temperature values from all grids are included in the density estimation process. Densities are estimated using the Kernel Density Estimation method.

REFERENCES

[1.](https://www.zotero.org/google-docs/?ReGjOH) [Cornes, R. C., Van Der Schrier, G., Van Den Besselaar, E. J. M. & Jones, P. D. An Ensemble Version of the E‐OBS Temperature and Precipitation Data Sets. *J. Geophys. Res. Atmospheres* **123**, 9391–9409, https://doi.org/10.1029/2017JD028200 (2018).](https://www.zotero.org/google-docs/?ReGjOH)
